# Beyond diagonal noise: A better predator-prey modeling framework with cross-covariance

**DOI:** 10.1371/journal.pone.0350127

**Published:** 2026-05-27

**Authors:** Jiguang Yu, Louis Shuo Wang

**Affiliations:** 1 College of Engineering, Boston University, Boston, Massachusetts, United States of America; 2 Department of Mathematics, University of Tennessee, Knoxville, Tennessee, United States of America; University of Dhaka, BANGLADESH

## Abstract

The introduction of stochasticity into continuous ecological models frequently relies on phenomenological, diagonal diffusion terms that lack a rigorous microscopic basis. We demonstrate that this standard practice fundamentally misrepresents the geometry of demographic fluctuations. By deriving a stochastic Rosenzweig–MacArthur model directly from an integer-valued, Bernoulli-coupled continuous-time Markov chain, we isolate the exact diffusion covariance structure dictated by event stoichiometry. We mathematically prove that coupled predation–conversion events inherently generate a structurally negative predator–prey cross-covariance, exposing the severe mathematical and biological limitations of standard diagonal-noise approximations. Furthermore, we resolve a persistent ambiguity in stochastic population modeling by explicitly formalizing the bifurcation between open-domain formulations (for survival-conditioned interior dynamics) and absorbed formulations (for extinction-permitting dynamics). To rigorously support this distinction, we develop a tailored two-stage Lyapunov well-posedness architecture that separates non-explosion criteria from boundary-barrier positivity invariance. By bridging microscopic event stoichiometry with macroscopic boundary-degenerate diffusions, this work replaces ad hoc noise constructs with a definitive, mathematically exact template for covariance-consistent and boundary-aware ecological modeling.

## Introduction

Predator–prey interactions constitute one of the foundational subjects of mathematical ecology. In particular, the Rosenzweig–MacArthur (R–M) model with Holling type II functional response remains a central benchmark for studying coexistence, oscillatory dynamics, and the paradox of enrichment [1–4]. In the deterministic setting, the R–M model exhibits a rich bifurcation structure. As the prey carrying capacity increases, the stable coexistence equilibrium loses stability through a Hopf bifurcation, which produces sustained limit cycles and increases the risk of low-density excursions. This mechanism, in which enrichment destabilizes coexistence, has attracted sustained attention in both ecological theory and applied population biology [5–7].

For finite populations, however, the deterministic ordinary differential equation (ODE) is merely a mean-field approximation. Demographic stochasticity, which arises from randomness in individual birth, death, and predation events, introduces fluctuations that can fundamentally alter quantitative predictions. Even when the deterministic coexistence equilibrium is locally stable, demographic noise can generate quasi-cycles and facilitate noise-induced transitions toward extinction [8,9]. These phenomena are especially pronounced near the Hopf threshold, where the deterministic restoring force weakens and fluctuation amplification becomes large. A rigorous analysis of these dynamics requires a stochastic description that is both mechanistically faithful to the underlying individual-level events and mathematically tractable for asymptotic study.

In this paper, we develop precisely such a description. We begin with an integer-valued continuous-time Markov chain (CTMC) formulation of the R–M system that includes explicit Bernoulli-coupled predation–conversion events. From this formulation, we derive a chemical master equation (CME) and, subsequently, a demographic diffusion approximation in the form of a chemical Langevin equation (CLE). Crucially, the drift and covariance of this CLE are strictly dictated by event stoichiometry and channel intensities. A central mathematical consequence of this derivation is that the mechanistically correct diffusion covariance is inherently non-diagonal: coupled predation–conversion events induce a structurally negative predator–prey cross-covariance. This geometric feature is absent from the phenomenological diagonal or multiplicative noise constructions that dominate the literature. We prove that this cross-covariance is a direct algebraic consequence of the stoichiometric coupling between prey loss and predator gain within the same demographic event, rather than an ad hoc modeling embellishment.

Beyond the mechanistic derivation of the noise structure, a primary aim of this work is to address a persistent source of ambiguity in stochastic predator–prey modeling. This ambiguity arises from the routine conflation of two distinct diffusion formulations built from the same local stochastic differential equation (SDE) coefficients. We establish that an open-domain formulation on the interior $(0,\infty {)}^{2}$ provides the appropriate framework for studying interior coexistence dynamics. This framework captures fluctuation geometry near equilibrium, survival-conditioned statistics, and local spectral structure. Conversely, an absorbed formulation, in which trajectories are frozen upon first contact with the boundary, provides the rigorous framework for extinction-permitting dynamics. This formulation addresses questions about extinction probabilities, mean persistence times, and quasi-stationary distributions, reflecting the irreversibility of CTMC extinction. These are not competing models for the same quantity; they answer fundamentally different biological questions. We argue that their distinction must be stated explicitly to avoid both modeling ambiguity and overreach in well-posedness claims.

Building upon the general literature on several topics presented in Literature review section in Appendix, the current work is structured to build our framework systematically, and the main contributions of this paper are as follows:

(C1) Mechanistic derivation of a full-covariance demographic diffusion. Starting from an integer-valued CTMC with Bernoulli-coupled predation–conversion events, we rigorously derive the CME and its associated CLE. This establishes that the diffusion covariance,
  $$\begin{array}{c} a(x)=\frac{1}{\Omega }\;S\;diag(f(x))\;S^{⊤}, \end{array}$$
  is strictly dictated by event stoichiometry and channel intensities, replacing phenomenological noise constructs with a purely mechanistic foundation.
(C2) Identification of a structurally negative predator–prey cross-covariance. We mathematically isolate the effect of coupled predation–conversion events, proving they inherently generate a strictly negative cross-covariance, *a*_12_(*x*) < 0, on the interior domain $D=(0,\infty {)}^{2}$. We demonstrate that drift-matched split-channel closures fail to capture this geometric feature. This result formalizes the limitations of diagonal-noise approximations and identifies regimes where they do not accurately represent the local fluctuation structure.
(C3) Formalization of the open-domain versus absorbed modeling bifurcation. We address a persistent ambiguity in stochastic ecological modeling by explicitly separating two global diffusion formulations built from the same local CME-consistent coefficients. We distinguish an open-domain formulation tailored for interior, survival-conditioned coexistence dynamics from an absorbed formulation designed for extinction-permitting dynamics, ensuring the mathematical framework aligns with the targeted biological observables.
(C4) A two-stage well-posedness architecture for boundary-degenerate diffusions. We develop a tailored analytical framework for the open-domain formulation that separates non-explosion from boundary avoidance. First, we establish maximal strong solvability without interior blow-up before boundary contact via a Lyapunov anti-explosion condition. Second, we establish positivity invariance as a distinct property dependent on an additional boundary-barrier condition, preventing the tacit assumption of survival from being embedded in baseline well-posedness claims.

**Fig 1:**
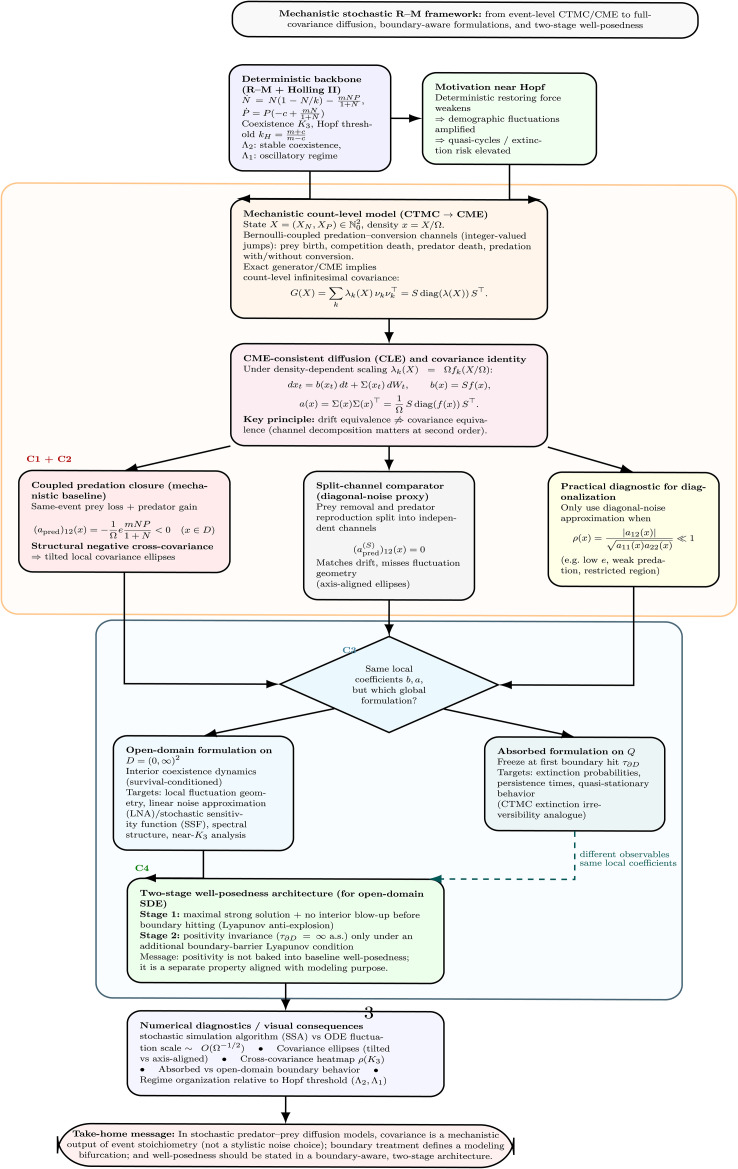
Mechanistic roadmap and modeling bifurcation for the stochastic R–M framework. The diagram summarizes the paper’s logic from the deterministic R–M backbone and Hopf organization, through a mechanistic CTMC/CME derivation of a full-covariance CLE, to the structural negative predator–prey cross-covariance induced by coupled predation–conversion. It then highlights the explicit modeling bifurcation between open-domain and absorbed diffusion formulations built from the same local coefficients, and the corresponding two-stage well-posedness architecture for the open-domain SDE.

Fig 1 presents the mechanistic roadmap and modeling bifurcation for the R–M model. The structure of this work is as follows. Methods section details the mathematical framework. Deterministic R–M backbone subsection records the deterministic R–M backbone. Mechanistic CTMC and CME subsection and CME-consistent covariance structure subsection develop the CTMC/CME formulation and the full-covariance diffusion derivation. Domain formulations subsection formalizes the open-domain and absorbed formulation distinction. Well-posedness subsection develops the two-stage well-posedness framework. Results section presents the numerical simulations that verify the boundary-aware, covariance-consistent modeling structure. Discussion section discusses implications and scope. Appendices provide full derivations of the CME-to-diffusion reduction and complete proofs of the well-posedness theorems.

## Methods

### Deterministic R–M backbone

This section records the deterministic R–M structure used as the background for the stochastic construction developed later.

We consider the nondimensional R–M predator–prey system with Holling type II predation [10]:


$$\begin{array}{c} \left\{ \begin{array}{c} \frac{dN}{dt}=N\;\left(1-\frac{N}{k}\right)-\frac{mNP}{1+N},\\ \frac{dP}{dt}=P\;\left(-c+\frac{mN}{1+N}\right), \end{array}\right.\;(N(t),P(t))\in Q:=\{(N,P):N\geq 0,\;P\geq 0\}, \end{array}$$ (1)

where *N* and *P* denote prey and predator densities, respectively, and *k* > 0, *m* > 0, *c* > 0 are the (scaled) carrying-capacity, predation/assimilation, and predator-mortality parameters. The biologically relevant closed state space is the positive quadrant *Q*, while the open quadrant


$$\begin{array}{c} D:=(0,\infty {)}^{2} \end{array}$$ (2)

will be the ambient domain for the open-domain diffusion formulation from Domain formulations subsection to Well-posedness subsection in Methods section. The vector field in (1) is smooth on *Q* (indeed on {N>−1, P∈R}).

We work with the standard reduced nondimensional R–M form in which the predator conversion factor is not written explicitly in the $e\frac{mNP}{1+N}$ term in the *P* equation in (1). In the mechanistic stochastic derivation (Mechanistic CTMC and CME subsection in Methods section), this corresponds to the normalization *e* = 1 when matching the reduced deterministic parameterization used throughout the analysis.

System (1) has three equilibria:


$$\begin{array}{c} K_{1}=(0,0), \end{array}$$ (3)

$$\begin{array}{c} K_{2}=(k,0), \end{array}$$ (4)

$$\begin{array}{c} K_{3}=(N^{*},P^{*})=\left(\frac{c}{m-c},\;\frac{k(m-c)-c}{k(m-c{)}^{2}}\right). \end{array}$$ (5)

The coexistence equilibrium *K*_3_ lies in *D* if and only if


$$\begin{array}{c} m>c\;\text{and}\;k(m-c)>c. \end{array}$$ (6)

The first condition ensures that the predator per-capita growth term


$$\begin{array}{c} -c+\frac{mN}{1+N} \end{array}$$

can become nonnegative for sufficiently large prey density, while the second condition is exactly the positivity condition $P^{*}>0$. In the present paper, *K*_3_ serves as the deterministic coexistence reference state for the mechanistic stochastic modeling and the boundary-aware diffusion formulations.

Let


$$\begin{array}{c} f(N,P)=N\;\left(1-\frac{N}{k}\right)-\frac{mNP}{1+N},\;g(N,P)=P\;\left(-c+\frac{mN}{1+N}\right). \end{array}$$

The Jacobian matrix is


$$\begin{array}{c} J(N,P)=(\begin{array}{cc} 1-\frac{2N}{k}-\frac{mP}{(1+N{)}^{2}} & -\frac{mN}{1+N}\\ \frac{mP}{(1+N{)}^{2}} & -c+\frac{mN}{1+N} \end{array}). \end{array}$$ (7)

At the coexistence equilibrium *K*_3_, one has $\det J(K_{3})>0$ whenever $K_{3}\in D$, so local stability is determined by the trace. A direct calculation gives the classical enrichment-driven Hopf threshold [10]


$$\begin{array}{c} k_{H}=\frac{m+c}{m-c}. \end{array}$$ (8)

Hence *K*_3_ is locally asymptotically stable for *k* < *k*_*H*_ and unstable for *k* > *k*_*H*_, with a Hopf bifurcation at $k=k_{H}$. We primarily use (8) to organize parameter regimes


$$\begin{array}{c} \Lambda_{2}≔\{(m,c,k):k<k_{H}\},\;\Lambda_{1}≔\{(m,c,k):k>k_{H}\}. \end{array}$$ (9)

**Fig 2:**
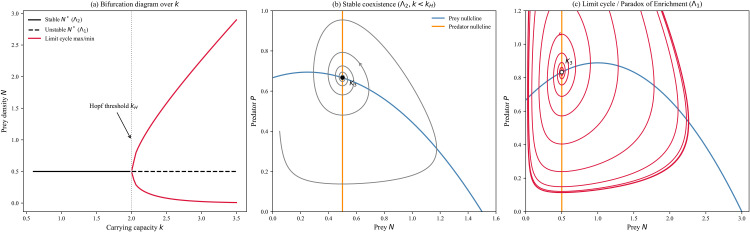
Deterministic R–M backbone and the Hopf bifurcation. **(a)** Bifurcation diagram over the carrying capacity ***k*.** The coexistence equilibrium *K*_3_ loses stability at the Hopf threshold *k*_*H*_, partitioning the parameter space into the stable regime $\Lambda_{2}$ and the oscillatory regime $\Lambda_{1}$. Solid curves denote the extrema of the stable limit cycle, and dashed lines denote the unstable equilibrium. **(b)** Phase portrait in the stable regime $\Lambda_{2}$ (*k* < *k*_*H*_). The predator nullcline (vertical) intersects the prey nullcline (humped) to the right of its peak, resulting in trajectories spiraling inward to a stable coexistence state *K*_3_. **(c)** Phase portrait in the oscillatory regime $\Lambda_{1}$ (*k* > *k*_*H*_). Enrichment shifts the intersection to the left of the peak, destabilizing *K*_3_ and generating a stable limit cycle that drives large-amplitude, low-density excursions.

Fig 2 illustrates the bifurcation diagram in the carrying capacity, and the phase portraits for the two regimes $\Lambda_{2}$ (stable coexistence) and $\Lambda_{1}$ (unstable oscillation), showing the Hopf bifurcation.

### Mechanistic CTMC and CME

We now formulate an integer-valued CTMC for the R–M predator–prey system and record the associated CME. Let


$$\begin{array}{c} X(t)=(X_{N}(t),X_{P}(t){)}^{⊤}\in N_{0}^{2} \end{array}$$

denote prey and predator counts in a well-mixed population of system size Ω (e.g., habitat volume or scaling parameter), and define the corresponding density process


$$\begin{array}{c} x(t)=\frac{X(t)}{\Omega }=(N(t),P(t){)}^{⊤}\in R_{+}^{2}. \end{array}$$

Because the exact CTMC is integer-valued, all jump increments in *X* must lie in $Z^{2}$. In particular, a compact predation–conversion increment of the form $(-1,e{)}^{⊤}$ with e∈(0,1] is not an admissible exact CTMC jump unless e∈N. We therefore formulate the CTMC using integer-valued channels (with Bernoulli conversion), and only later use an effective $(-1,e{)}^{⊤}$ increment as a density-level diffusion closure in CME-consistent covariance structure subsection in Methods section. This distinction is essential for mechanistic consistency.

We use the following five integer-valued event channels:


$$\begin{array}{c} \begin{array}{ccc} & R_{1}:\;X_{N}⟶X_{N}+1 & \text{(prey birth)},\\ & R_{2}:\;X_{N}⟶X_{N}-1 & \text{(prey competition death)},\\ & R_{3}:\;X_{P}⟶X_{P}-1 & \text{(predator death)},\\ & R_{4}^{\left(B\right)}:\;(X_{N},X_{P})⟶(X_{N}-1,\;X_{P}+1) & \text{(predation with successful conversion)},\\ & R_{5}^{\left(B\right)}:\;(X_{N},X_{P})⟶(X_{N}-1,\;X_{P}) & \text{(predation without conversion)}. \end{array} \end{array}$$

This is equivalent to a predation encounter process followed by Bernoulli conversion with success probability e∈(0,1]. The stoichiometric increments are


ν1=(10),ν2=(−10),ν3=(0−1),ν4(B)=(−11),ν5(B)=(−10), (10)

and the corresponding stoichiometric matrix is


$$\begin{array}{c} S_{B}:=[\nu_{1}\;\nu_{2}\;\nu_{3}\;\nu_{4}^{\left(B\right)}\;\nu_{5}^{\left(B\right)}]\in R^{2\times 5}. \end{array}$$ (11)

We adopt density-dependent (Kurtz-type) propensities


$$\begin{array}{c} \lambda_{k}(X)=\Omega \;f_{k}(x),\;x=\frac{X}{\Omega }, \end{array}$$ (12)

with intensity functions


$$\begin{array}{c} f_{1}(x)=N,\;f_{2}(x)=\frac{N^{2}}{k},\;f_{3}(x)=cP, \end{array}$$ (13)

$$\begin{array}{c} f_{4}^{\left(B\right)}(x)=e\;\frac{mNP}{1+N},\;f_{5}^{\left(B\right)}(x)=(1-e)\;\frac{mNP}{1+N}. \end{array}$$ (14)

Let $f_{B}=(f_{1},f_{2},f_{3},f_{4}^{\left(B\right)},f_{5}^{\left(B\right)}{)}^{⊤}$. Then the deterministic drift induced by the CTMC is


$$\begin{array}{c} b_{B}(x)=S_{B}f_{B}(x)=(\begin{array}{c} N\;\left(1-\frac{N}{k}\right)-\frac{mNP}{1+N}\\ -cP+e\;\frac{mNP}{1+N} \end{array}). \end{array}$$ (15)

This is the R–M drift with explicit conversion efficiency *e*.

In the current mechanistic CTMC/CME formulation, e∈(0,1] enters the predator gain term but not the prey loss term. To maintain exact consistency with the deterministic backbone (1) used in Deterministic R–M backbone subsection in Methods section, we perform the main stochastic analysis under the normalization


$$\begin{array}{c} e=1. \end{array}$$

The mechanistic derivations in this section are written for general *e* where useful (to expose the origin of covariance structure), and the specialization *e* = 1 is imposed when matching the reduced R–M parameterization used from Domain formulations subsection in Methods section to Results section.

For later comparison, it is useful to note that one can construct an alternative integer-valued CTMC with separate channels for prey removal and predator reproduction, using increments $(-1,0{)}^{⊤}$ and $(0,1{)}^{⊤}$ with rates proportional to $\frac{mNP}{1+N}$ and $e\;\frac{mNP}{1+N}$, respectively. This split-channel construction reproduces the same deterministic drift (15) but changes the count-level and diffusion-level covariance structure. In particular, it removes the same-event predator–prey coupling in the predation contribution and will therefore serve as a diagonal-noise comparator in CME-consistent covariance structure subsection in Methods section, not as the primary mechanistic baseline.

We formulate the CME. Let p(X,t)=P(X(t)=X), $X\in N_{0}^{2}$. For a finite family of channels with increments $\nu_{k}$ and propensities $\lambda_{k}(X)$, the CME is


$$\begin{array}{c} \frac{\partial }{\partial t}p(X,t)=\sum_{k=1}^{K}[\lambda_{k}(X-\nu_{k})\;p(X-\nu_{k},t)-\lambda_{k}(X)\;p(X,t)]. \end{array}$$ (16)

In the present Bernoulli-coupled model, *K* = 5, the increments are those in (10), and the propensities are given by (12)–(14). We use (16) primarily as the exact count-level probabilistic description from which moment identities and the diffusion covariance inherit their structure.

The associated CTMC generator acting on test functions $\varphi :N_{0}^{2}\to R$ is


$$\begin{array}{c} (A\varphi )(X)=\sum_{k=1}^{K}\lambda_{k}(X)\;(\varphi (X+\nu_{k})-\varphi (X)). \end{array}$$ (17)

As recalled in From the CME to the diffusion approximation section in Appendix, this yields both the count-scale drift increment


$$\begin{array}{c} \sum_{k=1}^{K}\lambda_{k}(X)\;\nu_{k} \end{array}$$

and the exact infinitesimal covariance (quadratic variation density) in count variables:


$$\begin{array}{c} G(X)=\sum_{k=1}^{K}\lambda_{k}(X)\;\nu_{k}\nu_{k}^{⊤}. \end{array}$$ (18)

Equivalently, in stoichiometric form,


$$\begin{array}{c} G(X)=S\;diag\;(\lambda (X))\;S^{⊤}, \end{array}$$ (19)

for the chosen channel representation (S,λ). This identity is exact at the CTMC level and already shows that second-order structure depends on event stoichiometry, not only on the drift.

The key implication of (18)–(19) is that, under density scaling x=X/Ω with $\lambda_{k}(X)=\Omega f_{k}(x)$, the diffusion covariance in the demographic-noise approximation is inherited directly from the count-level event structure. In CME-consistent covariance structure subsection in Methods section, we make this explicit through the unified formula


$$\begin{array}{c} a(x)=\frac{1}{\Omega }\;S\;diag(f(x))\;S^{⊤}, \end{array}$$

and use it to compare Bernoulli-coupled, effective coupled $(-1,e{)}^{⊤}$, and split-channel closures. This is where the structural negative predator–prey cross-covariance appears transparently.

### CME-consistent covariance structure

Let *S* denote a chosen stoichiometric matrix and *f*(*x*) the associated vector of density-level intensities. Under the density-dependent scaling $\lambda_{k}(X)=\Omega f_{k}(X/\Omega )$, the chemical Langevin diffusion approximation for the density process $x_{t}=X_{t}/\Omega$ takes the Itô form


$$\begin{array}{c} dx_{t}=b(x_{t})\;dt+\Sigma (x_{t})\;dW_{t}, \end{array}$$ (20)

with drift


$$\begin{array}{c} b(x)=Sf(x), \end{array}$$ (21)

and diffusion covariance


$$\begin{array}{c} a(x)≔\Sigma (x)\Sigma (x{)}^{⊤}=\frac{1}{\Omega }\;S\;diag\;(f(x))\;S^{⊤}. \end{array}$$ (22)

Equation (22) is the density-scale counterpart of the exact CTMC infinitesimal covariance identity in counts (Mechanistic CTMC and CME subsection in Methods section); see From the CME to the diffusion approximation section in Appendix for the derivation.

To isolate the predation contribution across different closures, we write


$$\begin{array}{c} f_{pred}(x):=\frac{mNP}{1+N},\;x=(N,P)\in D=(0,\infty {)}^{2}. \end{array}$$ (23)

Below we compare three drift-compatible predation closures (up to parameterization): (i) the exact Bernoulli-coupled CTMC mechanism, (ii) an effective diffusion-level coupled channel $(-1,e{)}^{⊤}$, and (iii) a split-channel comparator. All three closures reproduce the same first-order drift, but they differ at the level of quadratic variation and therefore in their diffusion covariance structure.

For the Bernoulli-coupled predation channels $R_{4}^{\left(B\right)}$ and $R_{5}^{\left(B\right)}$ from Mechanistic CTMC and CME subsection in Methods section, the density-level predation covariance contribution is


$$\begin{array}{cc} a_{pred}^{\left(B\right)}(x) & =\frac{1}{\Omega }[f_{4}^{\left(B\right)}(x)\;\nu_{4}^{\left(B\right)}(\nu_{4}^{\left(B\right)}{)}^{⊤}+f_{5}^{\left(B\right)}(x)\;\nu_{5}^{\left(B\right)}(\nu_{5}^{\left(B\right)}{)}^{⊤}]\\ & =\frac{1}{\Omega }\;f_{pred}(x)(\begin{array}{cc} 1 & -e\\ -e & e \end{array}). \end{array}$$ (24)

Hence, the off-diagonal predation term is


$$\begin{array}{c} (a_{pred}^{\left(B\right)}{)}_{12}(x)=-\frac{1}{\Omega }\;e\;f_{pred}(x)=-\frac{1}{\Omega }\;e\;\frac{mNP}{1+N}. \end{array}$$ (25)

In particular, for x∈D and *e* > 0, this term is strictly negative. Biologically, it records the same-event negative correlation created by prey removal and predator gain in a predation encounter with successful conversion.

For compact diffusion notation, one may use a single effective predation channel at the density level:


νeff=(−1e),feff(x)=fpred(x). (26)

This effective closure preserves the deterministic drift, but it aggregates the discrete predation–conversion mechanism into one diffusion jump. As a result, the predator fluctuation intensity is altered at second order. This is not an exact integer-valued CTMC jump when e∉N, but it is a valid diffusion-level closure consistent with the drift. The corresponding predation covariance contribution is


$$\begin{array}{c} a_{pred}^{\left(eff\right)}(x)=\frac{1}{\Omega }\;f_{pred}(x)\;\nu_{eff}\nu_{eff}^{⊤}=\frac{1}{\Omega }\;f_{pred}(x)(\begin{array}{cc} 1 & -e\\ -e & e^{2} \end{array}). \end{array}$$ (27)

Therefore,


$$\begin{array}{c} (a_{pred}^{\left(eff\right)}{)}_{12}(x)=-\frac{1}{\Omega }\;e\;f_{pred}(x)=-\frac{1}{\Omega }\;e\;\frac{mNP}{1+N}<0\;(x\in D,\;e>0). \end{array}$$ (28)

Thus, the effective coupled closure preserves the same predation-induced cross-covariance sign and magnitude as the exact Bernoulli-coupled mechanism, but it does not preserve the predator variance. Indeed, the exact Bernoulli-coupled closure gives a predator variance contribution proportional to *e*, whereas the effective single-channel closure gives *e*^2^. This is a direct consequence of collapsing a discrete success/failure conversion process into one diffusion jump, since diffusion covariances scale with the square of the effective jump size. The distinction is retained for the general e∈(0,1), while the numerical baseline *e* = 1 makes them coincide.

Consider the split-channel predation representation with


ν4(S)=(−10),ν5(S)=(01),f4(S)(x)=fpred(x),f5(S)(x)=efpred(x).

Then the predation-related covariance contribution is


$$\begin{array}{c} a_{pred}^{\left(S\right)}(x)=\frac{1}{\Omega }[f_{4}^{\left(S\right)}(x)\;\nu_{4}^{\left(S\right)}(\nu_{4}^{\left(S\right)}{)}^{⊤}+f_{5}^{\left(S\right)}(x)\;\nu_{5}^{\left(S\right)}(\nu_{5}^{\left(S\right)}{)}^{⊤}]=\frac{1}{\Omega }\;f_{pred}(x)(\begin{array}{cc} 1 & 0\\ 0 & e \end{array}). \end{array}$$ (29)

Hence


$$\begin{array}{c} (a_{pred}^{\left(S\right)}{)}_{12}(x)=0. \end{array}$$ (30)

This representation is drift-compatible with the coupled mechanisms but removes the same-event predator–prey fluctuation coupling. For that reason, we treat it as a diagonal-noise comparator, not as the default mechanistic closure.

**Proposition 0.1 (Structural predation cross-covariance under coupled versus split closures).** *Let* $x=(N,P)\in D=(0,\infty {)}^{2}$ *and* e∈(0,1]*. For the predation intensity* $f_{pred}(x)=\frac{mNP}{1+N}$*, the predation contribution to the diffusion covariance satisfies:*

(i) *under the exact Bernoulli-coupled closure,*
  $$\begin{array}{c} (a_{pred}^{\left(B\right)}{)}_{12}(x)=-\frac{1}{\Omega }\;e\;f_{pred}(x)<0; \end{array}$$
(ii) *under the effective coupled* (−1, *e*) *closure,*
  $$\begin{array}{c} (a_{pred}^{\left(eff\right)}{)}_{12}(x)=-\frac{1}{\Omega }\;e\;f_{pred}(x)<0; \end{array}$$
(iii) *under the split-channel closure,*

$$\begin{array}{c} (a_{pred}^{\left(S\right)}{)}_{12}(x)=0. \end{array}$$

Proposition 0.1 implies that the drift equivalence does not imply covariance equivalence. Different channel representations can produce the same deterministic drift while yielding different diffusion covariances. Indeed, the drift depends on the first stoichiometric moments


$$\begin{array}{c} b(x)=\sum_{k}f_{k}(x)\;\nu_{k}, \end{array}$$

whereas the covariance depends on the second stoichiometric moments


$$\begin{array}{c} a(x)=\frac{1}{\Omega }\sum_{k}f_{k}(x)\;\nu_{k}\nu_{k}^{⊤}. \end{array}$$

Thus, channel decompositions that are indistinguishable at the ODE level may differ at the diffusion level. This is the precise mechanism behind the distinction between coupled predation closures (which produce predator–prey cross-covariance) and split-channel closures (which can suppress it).

**Fig 3:**
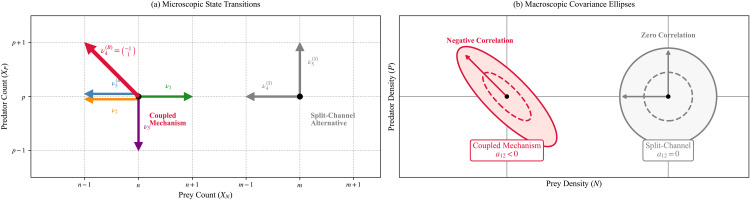
Mechanistic origin of the demographic diffusion covariance. **(a)** Microscopic state transitions on the count lattice. The exact Bernoulli-coupled mechanism strictly requires a diagonal jump vector $\nu_{4}^{\left(B\right)}$ (crimson) to represent a single predation–conversion event. The split-channel comparator falsely separates this into two independent, orthogonal jumps. **(b)** Macroscopic covariance geometry at the density scale. The diagonal jump in the coupled mechanism inherently tilts the local fluctuation geometry, generating a strictly negative predator–prey cross-covariance (*a*_12_ < 0). In contrast, the phenomenological split-channel closure produces an axis-aligned covariance ellipse (*a*_12_ = 0), failing to capture the true geometric structure of the demographic noise despite maintaining mean-field drift equivalence.

Within the class of drift-compatible predation closures considered above, the sign and presence of predator–prey cross-covariance at the diffusion level is determined by whether prey loss and predator gain occur in the same event channel. In particular, the negative off-diagonal term is not an ad hoc modeling embellishment; it is a stoichiometric consequence of coupled predation–conversion. Fig 3 shows the microscopic state transitions with arrows indicating the stoichiometric increments, and the macroscopic covariance ellipses with negative correlation tilting the fluctuation geometry.

**Remark 0.2 (Bernoulli-coupled exact CTMC vs. effective** $(-1,e{)}^{⊤}$ **closure).** *The exact Bernoulli-coupled CTMC closure and the effective* $(-1,e{)}^{⊤}$ *diffusion closure agree in deterministic drift and in the predation-induced cross-covariance magnitude*


$$\begin{array}{c} -\frac{1}{\Omega }\;e\;f_{pred}(x), \end{array}$$

*but they differ in the predator-variance contribution:*


$$\begin{array}{c} (a_{pred}^{\left(B\right)}{)}_{22}(x)=\frac{1}{\Omega }\;e\;f_{pred}(x),\;(a_{pred}^{\left(eff\right)}{)}_{22}(x)=\frac{1}{\Omega }\;e^{2}\;f_{pred}(x). \end{array}$$

*In this paper, the effective closure is used as a compact full-covariance diffusion representation, while the Bernoulli-coupled CTMC remains the exact integer-valued mechanistic foundation.*

For later reference, it is sometimes convenient to decompose the full covariance into non-predation and predation contributions:


$$\begin{array}{c} a(x)=a_{base}(x)+a_{pred}(x), \end{array}$$

where $a_{base}(x)$ collects prey birth, prey competition death, and predator death channels, and $a_{pred}(x)$ is one of


$$\begin{array}{c} a_{pred}^{\left(B\right)}(x),\;a_{pred}^{\left(eff\right)}(x),\;a_{pred}^{\left(S\right)}(x). \end{array}$$

Since the base channels in the present construction contribute only diagonal terms, the sign and magnitude of the full off-diagonal covariance *a*_12_(*x*) are determined entirely by the predation component. Therefore, Proposition 0.1 directly controls the sign of the full predator–prey cross-covariance in the coupled closures.

Since *a*(*x*) is symmetric positive semidefinite on $R_{+}^{2}$, it admits (non-unique) factorizations


$$\begin{array}{c} a(x)=\Sigma (x)\Sigma (x{)}^{⊤}. \end{array}$$ (31)

Two useful choices are:

(i) Event-based factorization that uses *K* independent Brownian drivers (one per channel):
$$\begin{array}{c} \Sigma_{K}(x)=\frac{1}{\sqrt{\Omega }}\;S\;\sqrt{diag(f(x))}\in R^{d\times K}, \end{array}$$
  which preserves channel semantics and is convenient for interpreting noise sources;
(ii) Minimal-dimensional square root (e.g., Cholesky): choose a measurable 2 × 2 square root $\Sigma_{2}(x)$ satisfying $\Sigma_{2}(x)\Sigma_{2}(x{)}^{⊤}=a(x)$, which is convenient for numerical simulation and for the formulation of the open-domain SDE.

These choices are equivalent in law at the level of the diffusion process defined by (20), provided they generate the same covariance matrix *a*(*x*).

**Assumption 0.3** (Diagonal-noise as a controlled approximation: small relative cross-covariance). *Let* R⊂D *be a state region of interest. A diagonal-noise approximation is considered only in regimes where*


$$\begin{array}{c} \rho (x):=\frac{\left|a_{12}(x)\right|}{\sqrt{a_{11}(x)\;a_{22}(x)}}\ll 1,\;x\in R. \end{array}$$ (32)

*That is, diagonalization is treated as a diagnostic approximation rather than a default mechanistic model.*

**Remark 0.4** (Relating small cross-covariance to ecological parameters). *Under the coupled closures, the cross-covariance term |a*_*12*_*(x)| is driven directly by the predation event rate, scaling as* $\frac{1}{\Omega }e\;f_{pred}(x)$*. Assuming a standard functional response* $f_{pred}(x)=\frac{mNP}{1+N}$ *(with maximum predation rate m), the condition* ρ(x)≪1 *in (32) translates directly into specific ecological parameter regimes where independent demographic processes dominate over coupled interaction events. A modeler might find a diagonal approximation accidentally valid in the following scenarios:*

(i) ***Low conversion efficiency (***e≪1***):*** *The predator requires consuming many prey items to produce a single offspring. This mechanically decouples the noise of a prey death from a predator birth, significantly shrinking the cross-covariance relative to the individual variances.*
(ii) **Weak maximum predation rate (***m***):** *If the predation rate m is small relative to the intrinsic growth or natural mortality parameters of the species, the interaction variance driven by* *f*_pred_(*x*) *is dwarfed by the diagonal demographic noise terms a*_*11*_*(x) and a*_*22*_*(x).*
(iii) **state restriction (***k***):** *If environmental carrying capacity k restricts N or P to remain uniformly small on* R*, the overall predation rate* *f*_pred_(*x*) *is suppressed.*

*When these conditions fail (e.g., highly efficient predators, strong predation pressure m, and limited resources k), diagonal-noise closures are expected to misrepresent second-order statistics, in particular* Cov(*N*, *P*) *and the geometry of local fluctuations.*

In the current work, the CTMC/CME formulation provides a microscopic description, whereas the diffusion (CLE) approximation yields a macroscopic limit. Thus, our framework establishes a principled bridge between scales, aligning conceptually with hybrid multiscale modeling approaches that couple discrete events with continuum dynamics [11,12].

### Domain formulations

The open-domain formulation is the diffusion model posed on the interior *D*, with initial condition $x_{0}\in D$:


$$\begin{array}{c} dx_{t}=b(x_{t})\;dt+\Sigma (x_{t})\;dW_{t},\;x_{0}\in D. \end{array}$$ (33)

Under the full-covariance coupled closure, the predation mechanism induces a negative off-diagonal term *a*_12_(*x*)<0 for x∈D, as shown in CME-consistent covariance structure subsection in Methods section. At this stage, (33) is a local SDE statement on an open set; the issue of boundary attainability is deferred to Well-posedness subsection in Methods section.

To model extinction-permitting dynamics, one uses an absorbed formulation based on the same local coefficients. Let


$$\begin{array}{c} \tau_{\partial D}:=\inf\{t\geq 0:\;x_{t}\notin D\} \end{array}$$ (34)

denote the first exit (boundary hitting) time from the interior for a local solution of (33). The absorbed process is then defined by freezing at the first boundary hit:


$$\begin{array}{c} x_{t}^{abs}:=\left\{ \begin{array}{cc} x_{t}, & t<\tau_{\partial D},\\ x_{\tau_{\partial D}}, & t\geq \tau_{\partial D}. \end{array}\right. \end{array}$$ (35)

Biologically, the boundary ∂D corresponds to loss of strict coexistence: *N* = 0 (prey extinction), *P* = 0 (predator extinction), or (0,0) (total extinction). The freezing rule in (35) is the diffusion-level analogue of the irreversibility of extinction in the underlying finite-population CTMC.

**Remark 0.5** (Why these are different questions (and why the distinction matters)). *The open-domain and absorbed formulations differ in at least three ways. First, they target different observables: interior fluctuation structure versus extinction/permanence statistics. Second, they differ in time-horizon emphasis: open-domain analyses are often local or survival-conditioned, whereas absorbed analyses directly encode rare-event accumulation and eventual boundary contact. Third, they correspond to different analogies with the exact CTMC: the absorbed formulation mirrors CTMC extinction irreversibility, while the open-domain formulation serves as a mathematically convenient interior approximation for coexistence dynamics.*

The distinction between the open-domain and absorbed formulations is not merely a technical distinction about how to close an SDE at the boundary. It corresponds to two different classes of biological observables. The open-domain formulation is appropriate when the empirical target is the fluctuation structure of populations that remain extant over the observational window. Examples include the amplitude and phase of predator–prey cycles, local covariance geometry around coexistence, quasi-cycles, stochastic amplification near a stable equilibrium, and survival-conditioned summaries of population variability. Such observables are central in empirical studies of population cycles and consumer–resource time series, where the goal is to explain persistent oscillatory abundance patterns rather than to estimate the first time at which one species disappears [13–15].

By contrast, the absorbed formulation is required when the empirical or management target is a boundary-hitting observable. In conservation applications, population viability analysis commonly estimates probabilities of extinction or quasi-extinction over a fixed horizon, distributions of persistence time, and mean or median time to extinction [16–18]. These quantities depend explicitly on whether and when a trajectory reaches a low-abundance or extinction boundary. A model that is conditioned to remain in the interior cannot answer such questions without changing the target quantity. Conversely, an absorbed model is not designed to describe the stationary or quasi-stationary geometry of surviving interior fluctuations after extinction events have been removed or conditioned away.

Thus, the modeling bifurcation reflects a biological difference in the question being asked. If the question concerns coexistence fluctuations among surviving populations, the relevant object is the interior law of the diffusion, often interpreted conditionally on non-extinction over the time scale of observation. If the question concerns extinction risk, persistence time, or quasi-extinction thresholds, the relevant object is the absorbed process. The same local drift and covariance coefficients may therefore support two globally different stochastic models because the empirical observables are different.

**Fig 4:**
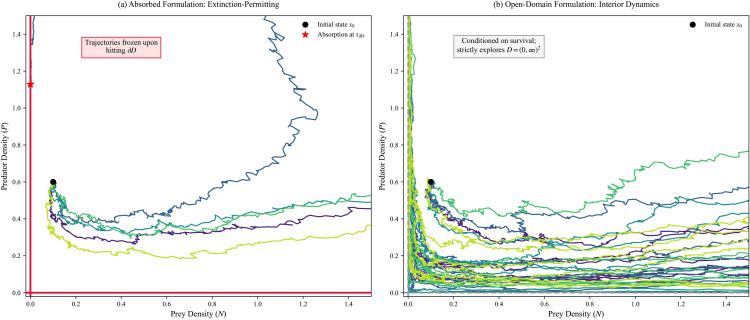
Visualizing the modeling bifurcation: Absorbed versus Open-Domain formulations. Both panels simulate the exact same local chemical Langevin drift and covariance parameters (Ω=150,k=5.5, a fluctuation-amplified regime) originating from the identical initial state *x*_0_. **(a)** The absorbed formulation, where trajectories are permanently frozen upon first boundary contact ($\tau_{\partial D}$), marked by red stars. This is the mathematically appropriate framework for evaluating extinction probabilities and mean persistence times. **(b)** A numerical proxy for the open-domain formulation, where trajectories are conditioned to survive and continuously explore the interior domain $D=(0,\infty {)}^{2}$. This framework captures the geometry of quasi-stationary interior fluctuations but must not be conflated with the extinction-permitting dynamics strictly captured in **(a)**.

Fig 4 contrasts the representative trajectories in the open domain $D=(0,\infty {)}^{2}$ under the open-domain formulation, and those in the first quadrant $Q=[0,\infty {)}^{2}$ under the absorbed diffusion formulation.

### Well-posedness

We consider the Itô SDE (33) on the open domain *D*. For the R–M demographic diffusion considered here, the drift *b* and covariance *a* are smooth on *D*, while a chosen factorization Σ may fail to be globally Lipschitz near ∂D because demographic noise intensities vanish as N↓0 and/or P↓0. Nevertheless, on every precompact set K⋐D, the coefficients are locally bounded and (for the factorizations used in this paper) locally Lipschitz. This is the natural setting for localization and Lyapunov arguments.

Define the first exit (boundary hitting) time from the interior by (34) and freeze *x*_*t*_ according to the rule in (35). We put the detailed proof for Theorem 0.6 and Proposition 0.7 in Proofs for well-posedness section in Appendix.

**Theorem 0.6 (Maximal strong solution and no interior explosion before boundary hitting).** *Assume:*

*(A1)* ***Local regularity on compacts.*** *For every precompact set* K⋐D*, the coefficients b and* Σ *are Lipschitz and bounded on K.*

*(A2)* ***Lyapunov control at infinity.*** *There exist* $V_{\infty }\in C^{2}(D;[0,\infty ))$ *and a constant C > 0 such that*


$$\begin{array}{c} LV_{\infty }(x)\leq C(1+V_{\infty }(x)),\;x\in D, \end{array}$$ (36)

*where*


$$\begin{array}{c} LV(x)=\nabla V(x)\cdot b(x)+\frac{1}{2}tr\;(a(x)\nabla^{2}V(x)),\;a(x)=\Sigma (x)\Sigma (x{)}^{⊤}, \end{array}$$

*and* $V_{\infty }(x)\to \infty$ *whenever* ‖x‖→∞ *with* x∈D.

*Then for every* $x_{0}\in D$*, the SDE (33) admits a unique maximal strong solution*


$$\begin{array}{c} (x_{t}{)}_{0\leq t<\tau_{*}} \end{array}$$

*with lifetime* $\tau_{*}\in (0,\infty ]$*, and*


$$\begin{array}{c} P(\tau_{*}<\infty ,\;\tau_{*}<\tau_{\partial D})=0. \end{array}$$ (37)

*Equivalently, loss of well-posedness cannot occur in the interior of D; if* $\tau_{*}<\infty$*, the only possible obstruction is boundary hitting (or boundary approach), i.e.,*


$$\begin{array}{c} \tau_{*}=\tau_{\partial D}\;\text{a.s.} \end{array}$$ (38)

**Proposition 0.7** (Positivity invariance under a boundary-barrier Lyapunov condition). *Assume the hypotheses of Theorem 0.6. In addition, suppose there exists a function*


$$\begin{array}{c} V_{b}\in C^{2}(D;[0,\infty )) \end{array}$$

*such that:*

***(B1) Boundary blow-up (barrier property).***


$$\begin{array}{c} V_{b}(x)\to \infty \;\text{whenever }x\to \partial D\text{ within }D. \end{array}$$ (39)

***(B2) Localized generator bound.*** *There exists* $C_{b}>0$ *such that for every* *n* ≥ 1 *and all* $x\in K_{n}$,


$$\begin{array}{c} LV_{b}(x)\leq C_{b}(1+V_{b}(x)). \end{array}$$ (40)

*Then*


$$\begin{array}{c} P(\tau_{\partial D}<\infty )=0. \end{array}$$ (41)

*Consequently, the maximal strong solution is global and remains in D for all times:*


$$\begin{array}{c} P(x_{t}\in D\text{ for all }t\geq 0)=1. \end{array}$$ (42)

For better understanding, we apply Theorem 0.6 to our model. We note that the computations below are applicable to all e∈(0,1] cases, while we only take *e* = 1 to remain consistent with the current framework. Here,


$$\begin{array}{c} b(x)=(\begin{array}{c} N(1-\frac{N}{k})-\frac{mNP}{1+N}\\ -cP+\frac{mNP}{1+N} \end{array}),\;a(x)=\frac{1}{\Omega }(\begin{array}{cc} N+\frac{N^{2}}{k}+\frac{mNP}{1+N} & -\frac{mNP}{1+N}\\ -\frac{mNP}{1+N} & cP+\frac{mNP}{1+N} \end{array}). \end{array}$$

We take the Lyapunov function as $V(x)=U(x{)}^{\alpha }$ where $U(x)=1+N^{2}+P^{2}$ and α>2. Then, it is easy to see


$$\begin{array}{c} ℒV(x)=\left(N(1-\frac{N}{k})-\frac{mNP}{1+N}\right)\partial_{N}V(x)+\left(-cP+\frac{mNP}{1+N}\right)\partial_{P}V(x)\\ +\frac{1}{2\Omega }\left(N+\frac{N^{2}}{k}+\frac{mNP}{1+N}\right)\partial_{NN}V(x)-2\frac{mNP}{1+N}\partial_{NP}V(x)+\left(cP+\frac{mNP}{1+N}\right)\partial_{PP}V(x). \end{array}$$

Here,


$$\begin{array}{c} \partial_{N}V(x)=2\alpha NU(x{)}^{\alpha -1},\;\partial_{P}V(x)=2\alpha PU(x{)}^{\alpha -1},\\ \partial_{NN}V(x)=2\alpha U(x{)}^{\alpha -1}+4\alpha (\alpha -1)N^{2}U(x{)}^{\alpha -2},\\ \partial_{PP}V(x)=2\alpha U(x{)}^{\alpha -1}+4\alpha (\alpha -1)P^{2}U(x{)}^{\alpha -2},\\ \partial_{NP}V(x)=4\alpha (\alpha -1)NPU(x{)}^{\alpha -2}. \end{array}$$

By elementary inequalities, e.g.,


$$\begin{array}{c} \frac{mNP}{1+N}\leq mP\leq \frac{mU(x)}{2},\;\max\{N,P,NP,N^{2},P^{2}\}\leq U(x), \end{array}$$

it is easy to find a constant C=C(α,m,c,k)>0 that satisfies


$$\begin{array}{c} ℒV(x)\leq C(1+V(x)),\;x\in D. \end{array}$$

Although the choice $V(x)=(1+N^{2}+P^{2}{)}^{\alpha }$ is primarily motivated by analytical convenience, it admits a natural ecological interpretation. Since *V*(*x*) is a strictly increasing function of $N^{2}+P^{2}$, it can be viewed as a radial abundance index for the predator–prey system, measuring the overall magnitude of the population state (*N*,*P*). In particular, $U(x)=1+N^{2}+P^{2}$ is comparable to the squared Euclidean distance from the extinction state (0,0), and hence *V*(*x*) provides a smooth proxy for the distance from extinction. Large values of *V*(*x*) correspond to configurations where at least one population is large, while small values correspond to states close to extinction. We emphasize that *V*(*x*) is not intended to represent a conserved ecological quantity such as total biomass *N* + *P*. Instead, it plays the role of a coercive control function that penalizes large excursions of either species. On comparable density scales, it can nevertheless be interpreted as a surrogate for aggregate biomass, while retaining the regularity and growth properties required for the Lyapunov analysis.

**Fig 5:**
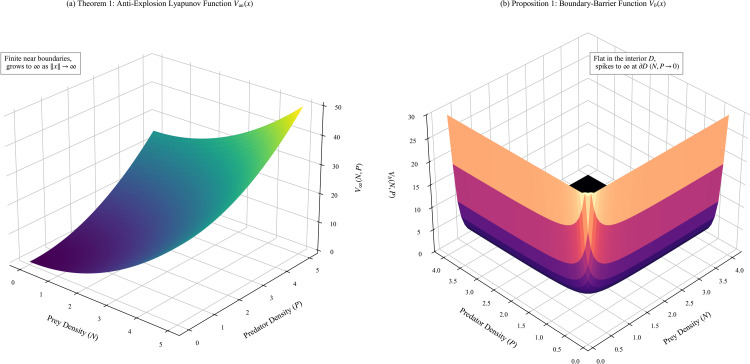
Geometric visualization of the two-stage Lyapunov well-posedness architecture. **(a)** The anti-explosion Lyapunov function *V*_∞_(*x*) (Theorem 0.6) acts as a geometric bowl that grows radially to infinity, strictly confining the stochastic trajectory from escaping to infinite states without restricting its boundary approach. **(b)** The boundary-barrier Lyapunov function *V*_*b*_(*x*) (Proposition 0.7) acts as a steep geometric cliff. The surface is extremely flat within the interior domain $D=(0,\infty {)}^{2}$, permitting natural quasi-stationary fluctuations, but spikes asymptotically to infinity precisely at the boundary faces *N* = 0 and *P* = 0, theoretically repelling the trajectory and ensuring positivity invariance almost surely.

Fig 5 visualizes the mathematical mechanisms for the prevention of interior blow-up and boundary hitting that underlies Theorem 0.6 and Proposition 0.7.

Demographic diffusions often invite comparison with one-dimensional square-root diffusions, where Feller boundary classification [19,20] gives explicit criteria for boundary attainability in terms of drift–diffusion balance. That intuition is useful but incomplete here. In the present two-dimensional predator–prey diffusion:

(i) drift and diffusion depend on both coordinates via nonlinear interaction terms;
(ii) the covariance need not be diagonal (indeed, the mechanistic full-covariance closure produces *a*_12_(*x*)<0 in *D*);
(iii) the boundary has multiple faces (*N* = 0, *P* = 0) with distinct biological meanings.

Accordingly, a literal one-dimensional Feller criterion does not transfer directly to the coupled two-dimensional setting. The barrier-based Lyapunov framework above is designed precisely to handle this multivariate, boundary-degenerate structure without forcing an oversimplified 1D analogy.

## A step-by-step guide for applyivng the framework to other ecological systems

Although the R–M predator–prey system provides the main worked example in this paper, the framework is not specific to two species or to Holling type II predation. The essential object is not the deterministic vector field alone, but the event-level representation from which the drift and covariance are derived. We therefore summarize the transferable procedure as a five-step workflow.

**Step 1: Identify the integer-valued demographic events.** The starting point is a list of biologically meaningful events that change integer population counts. These may include births, natural deaths, competition-induced deaths, predation events, conversion or reproduction events, immigration, harvesting, or disease transmission. Each event must be formulated as an admissible jump in the count process. For a system with species counts


$$\begin{array}{c} X(t)=(X_{1}(t),\ldots ,X_{d}(t){)}^{⊤}\in N_{0}^{d}, \end{array}$$

each event changes the state by an integer vector. This step is where biological mechanism enters the model most directly. In particular, if one ecological event simultaneously decreases one species and increases another, such as predation with conversion, then this coupling should be represented as a single joint event rather than as two independent noise sources.

For example, in a three-species food chain with basal prey *X*_1_, intermediate predator *X*_2_, and top predator *X*_3_, one may include prey birth, prey competition death, intermediate-predator death, top-predator death, predation of *X*_1_ by *X*_2_, and predation of *X*_2_ by *X*_3_. If predation is coupled to conversion, the corresponding count-level jumps include vectors such as


$$\begin{array}{c} (-1,+1,0{)}^{⊤}\;\text{and}\;(0,-1,+1{)}^{⊤}. \end{array}$$

These joint events automatically encode negative covariance between resource and consumer fluctuations.

In a two-species competition model, by contrast, typical demographic events might include intrinsic births $(+1,0{)}^{⊤}$, $(0,+1{)}^{⊤}$, intrinsic deaths $(-1,0{)}^{⊤}$, $(0,-1{)}^{⊤}$, and competition-induced deaths $(-1,0{)}^{⊤}$, $(0,-1{)}^{⊤}$ with rates depending on both species densities. Unless a single event changes both species simultaneously, the demographic covariance may remain diagonal even though the drift is dynamically coupled through the rates.

**Step 2: Define the stoichiometric matrix and channel intensities.** Once the event list is fixed, assign to each event *k* a stoichiometric increment


$$\begin{array}{c} \nu_{k}\in Z^{d} \end{array}$$

and collect the increments in the stoichiometric matrix


$$\begin{array}{c} S=[\nu_{1}\;\nu_{2}\;\cdots \;\nu_{K}]\in Z^{d\times K}. \end{array}$$

Next, specify density-dependent propensities


$$\begin{array}{c} \lambda_{k}(X)=\Omega f_{k}(X/\Omega ), \end{array}$$

where *f*_*k*_(*x*) is the density-level event intensity and Ω is the system-size parameter. This step separates the biological event mechanism from the scaling approximation. The increments $\nu_{k}$ describe what happens when an event occurs; the functions *f*_*k*_ describe how often those events occur.

For the three-species food-chain example, a coupled predation event of the intermediate predator on the basal prey may have


$$\begin{array}{c} \nu_{pred,12}=(-1,+1,0{)}^{⊤},\;f_{pred,12}(x)=\frac{m_{12}x_{1}x_{2}}{1+h_{12}x_{1}}, \end{array}$$

or another functional response appropriate to the system. A top-predator conversion event may similarly have


$$\begin{array}{c} \nu_{pred,23}=(0,-1,+1{)}^{⊤}. \end{array}$$

The specific functional response can change, but the covariance construction is unchanged.

**Step 3: Derive both the drift and the covariance from the same event representation.** The deterministic drift and the demographic diffusion covariance are then obtained from the same stoichiometric data:


$$\begin{array}{c} b(x)=Sf(x),\;a(x)=\frac{1}{\Omega }S\mathrm{diag}(f(x))S^{⊤}. \end{array}$$

This is the central methodological step. It ensures that the stochastic model is not obtained by adding phenomenological noise to an ODE, but by carrying the integer-valued event structure to the diffusion approximation.

This step is also where covariance geometry is determined. A joint event with increment $(-1,+1,0{)}^{⊤}$ contributes


$$\begin{array}{c} \frac{1}{\Omega }f_{pred,12}(x)(\begin{array}{ccc} 1 & -1 & 0\\ -1 & 1 & 0\\ 0 & 0 & 0 \end{array}) \end{array}$$

to the covariance matrix. Hence the basal prey and intermediate predator acquire a negative cross-covariance contribution. Similarly, a joint event $(0,-1,+1{)}^{⊤}$ contributes a negative cross-covariance between the intermediate and top predator. Thus, in a food chain, the full covariance matrix is generally banded or structured according to trophic event couplings, rather than diagonal.

For a competition model, if interspecific competition affects only the rates of single-species deaths, then the drift can be strongly coupled while the demographic covariance remains diagonal. This contrast illustrates the main principle of the present paper: drift coupling and noise coupling are different objects. Drift coupling is controlled by the dependence of *f*_*k*_(*x*) on the full state *x*, whereas covariance coupling is controlled by whether the same event channel changes multiple species simultaneously.

**Step 4: Specify the biologically appropriate domain formulation.** After the local coefficients *b*(*x*) and *a*(*x*) have been derived, the modeler must specify the global interpretation of the diffusion. For a *d*-species system, the natural interior domain is


$$\begin{array}{c} D=(0,\infty {)}^{d}. \end{array}$$

The open-domain formulation is appropriate when the target observables concern surviving coexistence dynamics: local covariance near an equilibrium, quasi-cycles, stochastic amplification, spectral structure, phase relationships, or survival-conditioned fluctuations. In contrast, the absorbed formulation is appropriate when the target observables are extinction probabilities, quasi-extinction probabilities, persistence-time distributions, or mean time to extinction. In conservation and population viability contexts, such boundary-hitting quantities are often the primary empirical or management target.

For a three-species food chain, different boundary faces have different biological meanings. The face *x*_3_ = 0 corresponds to loss of the top predator, *x*_2_ = 0 corresponds to collapse of the intermediate consumer, and *x*_1_ = 0 corresponds to basal-resource extinction. Absorbing all boundary faces may be appropriate when any species loss is treated as irreversible over the time scale of interest. Alternatively, one may define face-specific absorbing rules if the biological question distinguishes top-predator loss from total food-chain collapse. For a competition model, the boundary face *x*_*i*_ = 0 represents extinction of competitor *i*, and the absorbed formulation is the natural framework for coexistence probability, exclusion probability, or time-to-loss of a focal species.

**Step 5: Verify well-posedness and boundary behavior.** Finally, the modeler must verify that the resulting SDE is mathematically well posed for the intended formulation. The verification should be separated into two stages. First, local regularity on compact subsets of *D* and an anti-explosion Lyapunov function establish maximal strong solvability and rule out blow-up in the interior. A typical choice is a coercive abundance function such as


$$\begin{array}{c} V_{\infty }(x)={\left(1+\|x{\|}^{2}\right)}^{\alpha }, \end{array}$$

with α>2, followed by a generator estimate of the form


$$\begin{array}{c} LV_{\infty }(x)\leq C(1+V_{\infty }(x)). \end{array}$$

This step prevents unbounded population excursions from causing loss of the solution before any boundary is reached.

Second, if the intended model is an open-domain survival formulation, one must separately verify boundary non-attainment. This typically requires a boundary-barrier function *V*_*b*_ satisfying


$$\begin{array}{c} V_{b}(x)\to \infty \;\text{as }x\to \partial D \end{array}$$

together with a localized generator bound


$$\begin{array}{c} LV_{b}(x)\leq C_{b}(1+V_{b}(x)). \end{array}$$

This step should not be assumed automatically. It is a biological and mathematical modeling choice: if extinction-permitting dynamics are the target, then boundary hitting is not a pathology but the event of interest, and the absorbed formulation should be used instead.

This five-step workflow turns the present R–M analysis into a general method. The modeler first builds the integer-valued event system, then derives the diffusion covariance from stoichiometry, then selects the appropriate global domain interpretation, and finally verifies the well-posedness conditions required by that interpretation. The same procedure applies to predator–prey systems, food chains, competition models, host–parasitoid systems, epidemic–ecological models, and higher-dimensional food webs. What changes from model to model is the event list and the biological interpretation of the boundary; the covariance construction and domain logic remain the same.

## Results

This section provides illustrative numerical experiments supporting the structural results established from Mechanistic CTMC and CME subsection to Well-posedness subsection in Methods section. The emphasis is on visualizing the consequences of (i) the mechanistically induced diffusion covariance structure, in particular the predator–prey cross-covariance term, and (ii) the distinction between open-domain and absorbed formulations built from the same local coefficients.

All simulations use the nondimensional R–M system (1) with the Bernoulli-coupled predation–conversion mechanism of Mechanistic CTMC and CME subsection in Methods section. Unless otherwise stated, figures are generated from the exact CTMC (Gillespie SSA) or from Euler–Maruyama discretizations of the density-level diffusion under the covariance closures introduced in CME-consistent covariance structure subsection in Methods section. Throughout, we fix the baseline parameters


$$\begin{array}{c} m=1.5,\;c=0.4,\;e=1, \end{array}$$ (43)

and vary the carrying capacity *k* and system size Ω as indicated. Under (43), the Hopf threshold (8) is


$$\begin{array}{c} k_{H}=\frac{m+c}{m-c}=\frac{1.9}{1.1}\approx 1.727. \end{array}$$

### Covariance ellipses: Coupled versus split-channel closures

CME-consistent covariance structure subsection in Methods section established that drift-compatible closures can produce different diffusion covariances, and in particular that coupled predation–conversion induces a negative off-diagonal entry *a*_12_(*x*)<0, while the split-channel comparator yields $a_{12}^{\left(S\right)}(x)=0$ (Proposition 0.1). We visualize this local geometric difference at the coexistence equilibrium *K*_3_ through covariance ellipses.

**Fig 6:**
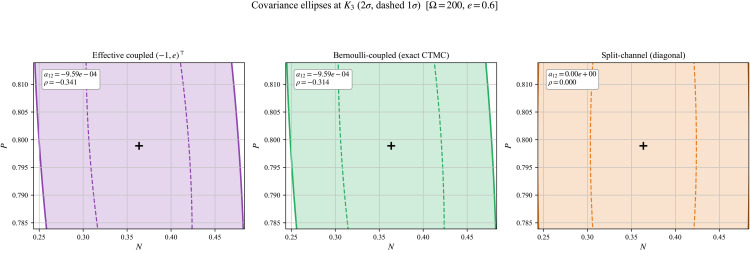
Covariance ellipses at *K*_3_ under three drift-compatible closures (Ω=200, *e* = 1). Left: effective coupled $(-1,e{)}^{⊤}$. Center: exact Bernoulli-coupled. Right: split-channel (diagonal comparator). Solid and dashed ellipses show 2σ and 1σ contours, respectively. The negative cross-covariance tilts the coupled-closure ellipses, while the split-channel ellipse is axis-aligned.

Fig 6 displays 1σ and 2σ covariance ellipses at *K*_3_ under three closures: (i) the effective coupled $(-1,e{)}^{⊤}$ closure (27), (ii) the exact Bernoulli-coupled closure (24), and (iii) the drift-compatible split-channel comparator (29). The two coupled closures produce tilted ellipses, reflecting the negative predator–prey cross-covariance, whereas the split-channel ellipse is axis-aligned. This provides a direct geometric signature of the structural sign result.

The panels also annotate the normalized cross-covariance ratio


$$\begin{array}{c} \rho (K_{3}):=\frac{a_{12}(K_{3})}{\sqrt{a_{11}(K_{3})\;a_{22}(K_{3})}}. \end{array}$$

As predicted by CME-consistent covariance structure subsection in Methods section, the Bernoulli-coupled and effective closures share the same off-diagonal term (hence the same sign and similar tilt direction), while differing in the predator variance entry *a*_22_; see Remark 0.2.

### Cross-covariance ratio across parameter space

To assess when a diagonal-noise approximation may be acceptable in practice (cf. Assumption 0.3 and Remark 0.4), we evaluate the normalized cross-covariance ratio $\rho (K_{3})$ across the (*m*,*k*) plane with *c* = 0.4 and *e* = 1 fixed.

**Fig 7:**
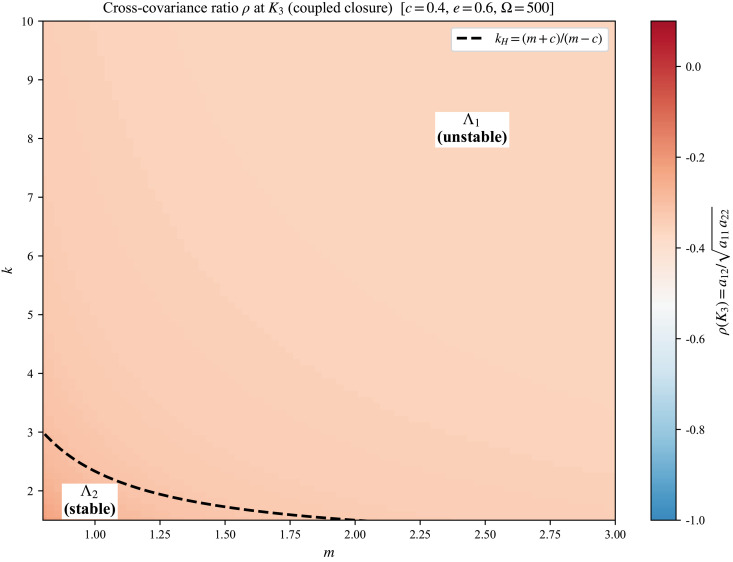
Heatmap of $\rho (K_{3})=a_{12}/\sqrt{a_{11}a_{22}}$ at coexistence under the effective coupled closure, scanned over (*m*,*k*) with *c* = 0.4, *e* = 1, Ω=500. The dashed curve is the Hopf threshold $k_{H}=(m+c)/(m-c)$. White regions indicate parameter values for which coexistence is infeasible.

Fig 7 shows $\rho (K_{3})$ under the effective coupled closure. The ratio is uniformly non-positive across the parameter region, in agreement with Proposition 0.1, and becomes more negative when *m*,*k* increase. The Hopf threshold is overlaid to indicate the partition of (*m*,*k*) parameter space into $\Lambda_{2}$ and $\Lambda_{1}$.

### Absorbed versus open-domain boundary behavior

Domain formulations subsection in Methods section introduced two distinct global interpretations built from the same local coefficients: an absorbed process for extinction-permitting dynamics and an open-domain formulation for interior dynamics. To visualize the modeling consequences of this distinction, we consider a parameter regime with amplified fluctuations (Ω=150, *k* = 5.5) and simulate representative sample paths over a finite horizon.

**Fig 8:**
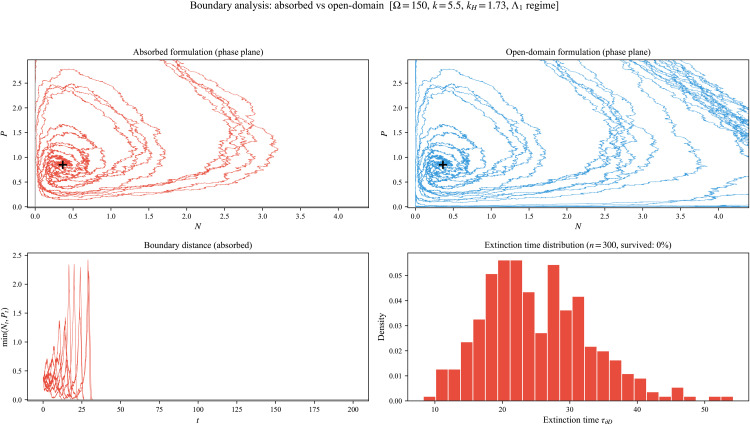
Boundary behavior at Ω=150, *k* = 5.5 (a fluctuation-amplified regime). **(a)** Absorbed formulation phase-plane paths. **(b)** Positivity-corrected Euler–Maruyama proxy for the open-domain interior-dynamics perspective. **(c)** Boundary-distance diagnostic $\min(N_{t},P_{t})$ for absorbed paths. **(d)** Empirical extinction-time distribution from *n* = 300 absorbed simulations.

Fig 8(a,d) uses the absorbed formulation, in which trajectories are frozen at first boundary contact, yielding an empirical extinction-time distribution. Fig 8(b) shows a positivity-corrected Euler–Maruyama proxy intended to visualize the interior-dynamics perspective associated with the open-domain formulation: when a discrete-time step produces a small sign violation, a numerical clipping near zero is applied to suppress step-size-induced boundary crossing behaviors. This numerical device is used only for visualization and should not be conflated with the continuous-time well-posedness statements proved in Well-posedness subsection in Methods section.

The contrast is nevertheless instructive. The absorbed formulation produces a nontrivial distribution of extinction times, while the open-domain proxy emphasizes continued interior fluctuation dynamics. Thus, although the two formulations share the same local drift and covariance in the interior, they answer different modeling questions and produce different observables.

### Parameter-regime visualization relative to the Hopf threshold

**Fig 9:**
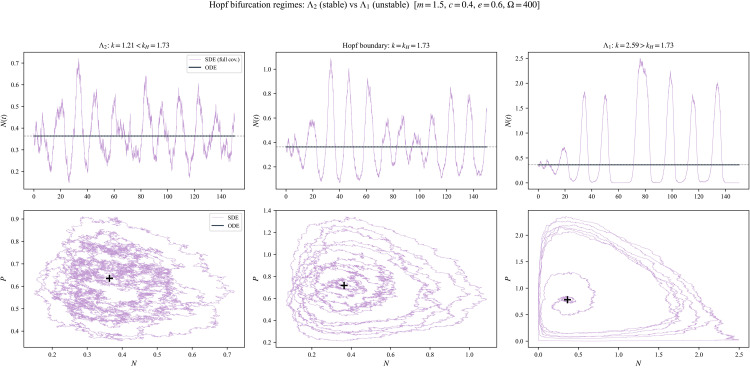
Trajectory regimes relative to the Hopf threshold at Ω=400. Left: $k=0.7\;k_{H}$ (inside $\Lambda_{2}$). Center: $k=k_{H}$ (threshold). Right: $k=1.5\;k_{H}$ (inside $\Lambda_{1}$). Top row: prey time series (diffusion vs. ODE). Bottom row: phase-plane trajectories.

Finally, we provide a brief visual comparison of stochastic trajectories across the parameter partition induced by the Hopf threshold (8). Fig 9 compares full-covariance diffusion simulations (with deterministic trajectories overlaid) for $k=0.7\;k_{H}$, $k=k_{H}$, and $k=1.5\;k_{H}$.

The purpose of this figure is primarily organizational: it illustrates that the partition into $\Lambda_{2}$ and $\Lambda_{1}$ corresponds to visibly different fluctuation regimes (stable-coexistence fluctuations, threshold-amplified oscillatory fluctuations, and noise-perturbed cycling, respectively).

Taken together, these numerical experiments reinforce the structural conclusions developed in the analytical sections. The covariance-ellipse visualizations provide a direct geometric manifestation of the mechanistically induced negative cross-covariance; the parameter-space scan shows that this structural sign persists across the parameter sweeps; and the boundary comparisons clarify that absorbed and open-domain formulations, though locally identical, encode distinct modeling objectives. The simulations, therefore, serve not as independent evidence but as visual diagnostics of the theoretical architecture: they illustrate how event-channel design, covariance structure, and boundary interpretation propagate from microscopic assumptions to macroscopic stochastic behavior.

## Discussion

This paper develops a mechanistically consistent stochastic framework for the R–M predator–prey model. It demonstrates that diffusion-level drift and covariance must be rigorously derived from an integer-valued CTMC/CME description rather than imposed directly at the SDE level. Ultimately, the value of this work lies not in proposing yet another stochastic variant of the R–M model, but in establishing a mathematically rigorous bridge linking: (i) microscopic event stoichiometry to macroscopic demographic covariance, (ii) open-domain survival to absorbed-boundary extinction, and (iii) boundary-degenerate local coefficients to a globally tailored well-posedness architecture.

### Mechanistic covariance structure as a primary result (Main result 1)

Our first principal contribution isolates the macroscopic consequences of microscopic event coupling. Starting from the Bernoulli-coupled predation–conversion CTMC, the diffusion covariance is strictly inherited through the CME-consistent identity


$$\begin{array}{c} a(x)=\frac{1}{\Omega }\;S\;diag(f(x))\;S^{⊤}. \end{array}$$

Within this framework, the predator–prey cross-covariance is not a tunable modeling embellishment: it is an unavoidable structural output of the stoichiometric coupling between prey loss and predator gain during predation–conversion events. In particular, the coupled closures systematically studied in CME-consistent covariance structure subsection in Methods section strictly produce a negative predation-induced off-diagonal term. This rigorously exposes the fallacy that drift equivalence implies covariance equivalence, demonstrating that diagonal-noise closures must be relegated to regime-specific approximations rather than treated as default mechanistic baselines.

### Boundary-aware formulation distinction as a modeling result (Main result 2)

The second principal result is the formal bifurcation of two global diffusion formulations built from the exact same local coefficients. One is an open-domain formulation on $D=(0,\infty {)}^{2}$, used strictly for interior, survival-conditioned dynamics. The other is an absorbed formulation, which allows for extinction-permitting dynamics. While routinely blurred or entirely ignored in the stochastic ecological literature, this distinction is both mathematically profound and biologically consequential. The open-domain formulation provides the precise analytical arena for coexistence fluctuations and local spectral geometry. Conversely, the absorbed formulation is the exclusive framework for evaluating extinction probabilities and mean persistence times, mirroring the irreversibility of CTMC extinction. By stating this bifurcation explicitly, our framework eradicates a pervasive source of modeling ambiguity.

### Two-stage well-posedness architecture as an analytical result (Main result 3)

The third principal result is the customized two-stage well-posedness architecture for the open-domain SDE. Well-posedness subsection in Methods section deliberately separates:

(i) maximal strong solvability together with non-explosion in the interior before boundary hitting, and
(ii) positivity invariance under an additional boundary-barrier Lyapunov condition.

This separation is not a mere technical bookkeeping device; it is a vital architectural safeguard. It explicitly aligns the mathematical theorems with the biological modeling distinction formalized in Domain formulations subsection in Methods section. Positivity invariance is a property that may hold in a specific open-domain analytical regime, but it is not a default mathematical right one can claim when the intended model is extinction-permitting. This formulation thereby avoids analytical overreach and provides a pristine mathematical template for other boundary-degenerate population diffusions.

### Scope and limitations

We contextualize these contributions within several recognized limitations. First, the diffusion model remains a second-order approximation to the exact CTMC/CME. It is therefore most robust in regimes where the system-size parameter Ω is sufficiently large and event counts over observational windows are strictly non-sparse. For severely small populations, integer-valued effects and immediate boundary absorption dominate, rendering exact CTMC/SSA descriptions as the mandatory baseline. Second, the present paper emphasizes exact structural derivation and well-posedness architecture rather than quantitative diffusion error bounds; no uniform-in-time approximation bound in Ω is pursued here. Third, the open-domain analysis is formulated at the abstract level of local coefficients and Lyapunov or barrier criteria. Verifying these conditions in highly specific or extreme parameter regimes may require bespoke analytical constructions beyond the generic bounding techniques presented.

### Generalizations and portability of the framework

Crucially, the analytical framework developed here is highly portable and extends well beyond the R–M model. The core covariance identity


$$\begin{array}{c} a(x)=\frac{1}{\Omega }\;S\;diag(f(x))\;S^{⊤} \end{array}$$

depends universally on event stoichiometry and intensity functions, completely agnostic to the specific Holling type II form. Thus, this exact blueprint can be deployed for alternative predator–prey mechanisms (e.g., ratio-dependent interactions) and seamlessly scaled to high-dimensional multi-species food-web models. More broadly, the integration of biological mechanisms, rigorous mathematical modeling, and threshold-driven organization reflects a portable analytical paradigm that extends to diverse biological systems beyond ecological interactions [21,22]. In every such extension, the same three modeling questions reappear. First, which event couplings mandate nontrivial cross-covariances? Second, which specific boundary faces represent biologically distinct extinction cascades? Third, which open-domain versus absorbed interpretations align with the target observables? This paper provides the foundational, reusable template for answering those questions explicitly.

## Conclusions

The central thesis of this paper is that demographic covariance in predator–prey diffusion models must be treated as a mechanistic output, not a stylistic choice. For the R–M system, predation–conversion coupling structurally mandates a negative predator–prey cross-covariance. This feature interacts fundamentally with boundary interpretation and stochastic well-posedness. We derive the diffusion directly from an integer-valued CTMC model, explicitly separate open-domain from absorbed formulations, and formalize a bespoke two-stage well-posedness architecture. Together, these steps establish a mathematically rigorous foundation for covariance-consistent, boundary-aware stochastic ecological modeling.

## Appendix

### Literature review

We present the necessary literature on which the current study is based. The present work connects several partially overlapping bodies of literature: (i) stochastic predator–prey modeling and its mechanistic foundations, (ii) demographic-noise amplified population oscillation, (iii) CME and chemical Langevin methods in population biology, and (iv) well-posedness and boundary analysis for diffusion processes arising in ecology and mathematical biology. We review each part in turn and identify the specific gaps that motivate the contributions of this paper.

#### Stochastic predator–prey models: Noise structures and their origins.

The introduction of noise into predator–prey systems has a long history. Early works by May [3] and Gard [23] studied stochastic Lotka–Volterra-type equations with environmental noise. They establish that random fluctuations can qualitatively alter or preserve persistence and coexistence depending on noise intensity. Subsequently, a large literature developed stochastic R–M models and related systems, using both Itô and Stratonovich SDE formulations with various noise structures [24–29].

A common modeling approach in this literature is to introduce multiplicative noise directly at the SDE level, typically by adding separate, independent Wiener processes to the prey and predator equations:


$$\begin{array}{c} dN=f(N,P)\;dt+\sigma_{1}N\;dW_{1},\;dP=g(N,P)\;dt+\sigma_{2}P\;dW_{2}, \end{array}$$

or variants thereof [30–34]. Such constructions are mathematically tractable and allow for clear persistence and extinction analyses. However, they remain fundamentally phenomenological rather than mechanistic because the noise intensities $\sigma_{1}$ and $\sigma_{2}$ are free parameters not derivable from event stoichiometry, and the independence of the two noise sources is imposed rather than justified. In particular, a predation event that simultaneously removes a prey individual and possibly adds a predator individual counts as a single demographic event. Its natural diffusion-level representation must involve a coupled, non-diagonal fluctuation increment rather than two independent ones. This mechanistic structure is inherently absent from diagonal noise models, and quantifying the severe consequences of this omission is a primary motivation for the present work.

A different strand of stochastic ecological modeling introduces noise through environmental (extrinsic) stochasticity rather than demographic (intrinsic) stochasticity [35–38]. Environmental noise models capture variation in parameters (e.g., carrying capacity or predation rate) driven by external fluctuations, and they also typically produce diagonal or block-diagonal noise structures. This paper focuses exclusively on demographic noise, arising from the discreteness of individual events in finite populations, and does not incorporate environmental noise. This distinction is standard in the theory of stochastic population models [39–42] and is strictly maintained throughout.

Several authors have emphasized the importance of the mechanistic derivation of noise structure in population models. Renshaw [43] and Allen [44] discuss CTMC formulations of birth–death–immigration processes and their diffusion limits. Naghnaeian and Del Vecchio [45] and Wang [46] develop moment approximations for interacting population models starting from the CME. Recently, Black and McKane [9] provide an accessible account of CME-based stochastic modeling in ecology, while Rogers et al. [47] use CME methods to study demographic fluctuations in multi-species systems. In these works, the importance of deriving the diffusion covariance from event stoichiometry rather than imposing it phenomenologically is recognized. However, the specific structural consequences for predator–prey covariance geometry and their implications for boundary behavior have not been systematically unraveled.

#### Quasi-cycles, stochastic amplification, and resonance near Hopf bifurcations.

The phenomenon of quasi-cycles in predator–prey and other ecological systems, which are stochastic oscillations in the stable regime driven by the amplification of demographic fluctuations, was clarified theoretically by McKane and Newman [8]. They used an individual-level model for a predator–prey system to demonstrate this behavior. Their analysis showed that demographic noise can produce power spectral peaks at a characteristic frequency, even when the deterministic equilibrium is stable. This result provides a mechanistic explanation for observed population oscillations without invoking nonlinear limit cycles. This result was extended to R–M-type systems by Barraquand [25], to spatial models by Butler and Goldenfeld [48,49], and to systems with multiple interacting species by Biancalani et al. [50]. Theoretical tools designed to capture these phenomena, such as the linear noise approximation (LNA), rely explicitly on the evaluation of the diffusion covariance matrix at equilibrium, $D_{*}=a(K_{3})$, to quantify these spectral peaks. However, without a bottom-up derivation, D_*_ is often misspecified as diagonal.

On the other hand, stochastic sensitivity analysis for nonlinear stochastic systems has been developed by Bashkirtseva and colleagues [51–53], including applications to mathematical ecology models. Their stochastic sensitivity function (SSF) formalism provides a geometric description of fluctuation amplification via Lyapunov-equation-based covariance analysis, and quantifies the local propensity for noise-induced transitions. Consequently, noisy precursors and early-warning signals near tipping points and bifurcations have attracted substantial interest as indicators of regime shifts in ecological systems [54–59]. Our rigorous identification of the negative cross-covariance provides the exact geometric input needed for these sensitivity and early-warning frameworks. This examines whether risk assessments of noise-induced extinction is fundamentally biased by diagonal-noise assumptions.

#### CME and Langevin methods in population biology.

The CME and the associated CLE derived via van Kampen system-size expansions are classical [60–62]. Gillespie [62] gives a rigorous derivation of the CLE from the CME under a “many firings” condition, yielding a diffusion approximation whose covariance is the density-rescaled version of the exact CTMC infinitesimal covariance. The key formula


$$\begin{array}{c} a(x)=\frac{1}{\Omega }\;S\;diag(f(x))\;S^{⊤} \end{array}$$

appears in this context as a direct mathematical consequence of the event stoichiometry and propensities; see also Kampen [60] and Elf and Ehrenberg [63].

Applications of the CME/CLE framework to ecological population models have been developed by Allen [44], Black and McKane [9], Ovaskainen and Meerson [40], and others. In predator–prey contexts specifically, Pineda-Krch et al. [64] apply Gillespie SSA to predator–prey systems; Alonso et al. [65] study demographic stochasticity and critical transitions using CME-based approximations. While the present paper builds upon this robust CLE/CME framework, it pivots from general moment closures to focus specifically on: (i) the mechanistic isolation of the non-diagonal covariance structure, (ii) the explicit comparison between coupled and split-channel CTMC closures, and (iii) the rigorous treatment of boundary behavior in the resulting diffusion.

#### Well-posedness and boundary analysis for population diffusions.

The well-posedness of SDEs arising in mathematical biology has been studied extensively. This is especially true for models with boundary-degenerate or state-constrained diffusion. For one-dimensional diffusions, Feller’s boundary classification [19,66] provides explicit conditions for the accessibility and attainability of boundaries in terms of drift–diffusion balance. Applications to population genetics (Wright–Fisher diffusion) are classical [67–69]. For square-root diffusions (CIR processes) [70], the Feller condition $2\kappa \theta \geq \sigma^{2}$ governs whether the boundary is accessible, and is widely used in financial mathematics and population modeling.

In two or higher dimensions, direct analogues of the Feller criterion are generally unavailable because boundary attainability depends on the full nonlinear drift–diffusion interaction and may differ across distinct boundary faces. Lyapunov function methods provide the standard replacement: non-explosion and positivity are established by finding a Lyapunov function satisfying a linear growth bound of the generator [71–73]. These methods have been used for stochastic Lotka–Volterra systems and related models [74–77], typically in the context of global well-posedness and long-time behavior under various noise conditions. A relevant model is the mathematical Leslie–Gower system, where a stochastic extension driven by demographic noise is established [78], but the well-posedness analysis is missing.

Our work adopts this Lyapunov perspective for the boundary-degenerate demographic diffusion arising from the R–M CTMC, but introduces a critical structural departure from the existing literature: a deliberate, two-stage separation of well-posedness claims. We first establish a maximal strong solution with no interior blow-up before boundary hitting (Theorem 0.6), and only then establish positivity invariance under an additional barrier condition (Proposition 0.7). This separation is explicitly motivated by the modeling bifurcation between open-domain and absorbed formulations. It also prevents positivity invariance, which is biologically relevant only in the open-domain formulation, from being inadvertently embedded as a baseline mathematical assumption in extinction-permitting modeling.

#### Harvesting and interaction.

Recent studies have further clarified how harvesting, heterogeneity, and transport mechanisms shape the fate of interacting populations. In a heterogeneous environment with harvesting, the competition of two species was shown to admit a well-posed PDE formulation, and the analysis revealed regimes in which coexistence, competitive exclusion, or even simultaneous extinction may occur, with numerical experiments supporting the theoretical conclusions [79]. A closely related work on two competing species in a nonhomogeneous habitat with harvesting established existence and uniqueness of solutions, identified conditions for coexistence and extinction, and compared the effects of different harvesting levels through both analysis and simulation [80]. More recently, a time-dependent advection-reaction-diffusion competition model with stocking and harvesting was analyzed for existence, uniqueness, and positivity, and the authors further developed fully discrete decoupled linearized schemes and showed numerically that appropriate stocking or harvesting can promote coexistence [81]. In a heterogeneous advective setting, another study investigated a two-species reaction–diffusion–advection model with unequal diffusion and advection rates, deriving local and global stability results and showing that the outcome of competition can depend on the ratio of transport rates [82]. Finally, a spatially distributed harvesting model with resource-based diffusion demonstrated that harvesting intensity and migration strategy can determine whether coexistence or competitive exclusion prevails, and it also provided estimates for harvesting regimes under which coexistence is possible [83]. Together, these works highlight that harvesting and transport effects can fundamentally alter persistence outcomes in structured ecological systems, thereby motivating mechanistically faithful stochastic formulations that track not only mean-field drift but also the associated covariance and boundary behavior.

Recent literature has continued to develop stochastic predator–prey theory from both general and mechanism-specific perspectives. A comprehensive treatment of deterministic, stochastic, and thermodynamic modeling of interacting species emphasized how birth and death, competition, consumption, and environmental fluctuations shape the stability and dynamics of ecological systems, thereby providing a broad foundation for stochastic ecological analysis [84]. Within predator–prey systems, one study incorporating additional food for the predator perturbed the prey growth rate and the predator death rate by Gaussian white noise, and established existence and uniqueness of a global positive solution together with extinction and persistence criteria [85]. Related work on a stochastic predator–prey model with additional food and a fear effect proved existence, uniqueness, boundedness, and uniform continuity of the global positive solution, while also deriving conditions for extinction and persistence under environmental noise [86]. Another study combined prey refuge, additional food for the predator, mutual interference, and white-noise perturbations, again proving positivity, boundedness, existence and uniqueness of global positive solutions, and identifying extinction and persistence regimes [87]. These works show that stochastic perturbations, additional food, fear, refuge, and environmental heterogeneity can substantially alter persistence and extinction outcomes, which motivates our mechanistic treatment of covariance structure and boundary-aware stochastic dynamics.

### From the CME to the diffusion approximation

This appendix records a standard but explicit route from a mechanistic CTMC, via the CME, to the diffusion approximation used in the main text. It also clarifies the origin of the covariance identity


$$\begin{array}{c} a(x)=\Sigma (x)\Sigma (x{)}^{⊤}=\frac{1}{\Omega }\;S\;diag(f(x))\;S^{⊤}, \end{array}$$ (44)

which underlies the full-covariance chemical Langevin formulation. At the count level, the corresponding infinitesimal covariance is


$$\begin{array}{c} G(X)=S\;diag(\lambda (X))\;S^{⊤}. \end{array}$$ (45)

For the predator–prey application in Mechanistic CTMC and CME subsection in Methods section, the key message is that the diffusion matrix is not an ad hoc modeling choice: it is inherited directly from the stoichiometry and propensities of the underlying CTMC/CME description.

#### CTMC formulation and the CME.

Let $X(t)\in N_{0}^{d}$ denote the vector of population counts (in the main text, *d* = 2 for prey and predator). Consider *K* reaction/event channels indexed by k=1,…,K, with stoichiometric increments $\nu_{k}\in Z^{d}$. Collect them in the stoichiometric matrix


$$\begin{array}{c} S=[\nu_{1}\;\nu_{2}\;\cdots \;\nu_{K}]\in Z^{d\times K}. \end{array}$$

When channel *k* fires at state *X*, the process jumps according to


$$\begin{array}{c} X⟼X+\nu_{k}. \end{array}$$

Let $\lambda_{k}(X)\geq 0$ be the propensity of channel *k* at state *X*. By definition, for a short interval [*t*,*t* + *dt*),


$$\begin{array}{c} P(\text{channel}k\text{fires in }[t,t+dt)|X(t)=X)=\lambda_{k}(X)\;dt+o(dt), \end{array}$$ (46)

and, up to *o*(*dt*), at most one event occurs.

Let p(X,t):=P(X(t)=X). Conditioning on what occurs during [*t*,*t* + *dt*), there are (up *t*o *o*(*dt*)) two contributions to *p*(*X*,*t* + *dt*):

(i) no event occurs and the sys*t*em was already in state *X*, contributing
  $$\begin{array}{c} p(X,t)(1-\sum_{k=1}^{K}\lambda_{k}(X)\;dt)+o(dt); \end{array}$$ (47)
(ii) exactly one event *k* occurs and moves the system from $X-\nu_{k}$ into *X*, contributing
  $$\begin{array}{c} \sum_{k=1}^{K}p(X-\nu_{k},t)\;\lambda_{k}(X-\nu_{k})\;dt+o(dt). \end{array}$$ (48)

Summing these terms, subtracting *p*(*X*,*t*), dividing by *dt*, and letting dt↓0 yields the CME:


$$\begin{array}{c} \frac{\partial }{\partial t}p(X,t)=\sum_{k=1}^{K}[\lambda_{k}(X-\nu_{k})\;p(X-\nu_{k},t)-\lambda_{k}(X)\;p(X,t)]. \end{array}$$ (49)

#### Generator and moment identities.

Let $\varphi :N_{0}^{d}\to R$ be a test function of suitable growth. Multiplying (49) by φ(X), summing over states, and using index shifts gives


$$\begin{array}{c} \frac{d}{dt}\;E[\varphi (X(t))]=E\;\left[(A\varphi )(X(t))\right], \end{array}$$ (50)

where the CTMC generator is


$$\begin{array}{c} (A\varphi )(X)=\sum_{k=1}^{K}\lambda_{k}(X)\;(\varphi (X+\nu_{k})-\varphi (X)). \end{array}$$ (51)

We compute the first moment (drift). Taking $\varphi (X)=X_{i}$ gives


$$\begin{array}{c} \varphi (X+\nu_{k})-\varphi (X)=(\nu_{k}{)}_{i}, \end{array}$$

hence


$$\begin{array}{c} \frac{d}{dt}\;E[X_{i}(t)]=E\;\left[\sum_{k=1}^{K}\lambda_{k}(X(t))\;(\nu_{k}{)}_{i}\right]. \end{array}$$ (52)

In vector form, this is the exact CTMC drift identity


$$\begin{array}{c} \frac{d}{dt}\;E[X(t)]=E\;\left[S\;\lambda (X(t))\right], \end{array}$$ (53)

where $\lambda (X)=(\lambda_{1}(X),\ldots ,\lambda_{K}(X){)}^{⊤}$.

We compute the second moment and quadratic variation structure. Taking $\varphi (X)=X_{i}X_{j}$ and using


$$\begin{array}{c} (X+\nu_{k}{)}_{i}(X+\nu_{k}{)}_{j}-X_{i}X_{j}=X_{i}(\nu_{k}{)}_{j}+X_{j}(\nu_{k}{)}_{i}+(\nu_{k}{)}_{i}(\nu_{k}{)}_{j}, \end{array}$$

we obtain


$$\begin{array}{c} \frac{d}{dt}\;E[X_{i}(t)X_{j}(t)]=E\;\left[\sum_{k=1}^{K}\lambda_{k}(X(t))((X(t){)}_{i}(\nu_{k}{)}_{j}+(X(t){)}_{j}(\nu_{k}{)}_{i}+(\nu_{k}{)}_{i}(\nu_{k}{)}_{j})\right]. \end{array}$$ (54)

The term $(\nu_{k}{)}_{i}(\nu_{k}{)}_{j}$ is the key source of infinitesimal covariance; in matrix form it appears as $\nu_{k}\nu_{k}^{⊤}$.

#### Infinitesimal covariance at the CTMC level.

Let ΔX:=X(t+dt)−X(t). Conditioning on *X*(*t*)=*X*, equation (46) implies


$$\begin{array}{c} E[\Delta X|X(t)=X]=\sum_{k=1}^{K}\lambda_{k}(X)\;\nu_{k}\;dt+o(dt), \end{array}$$

and


$$\begin{array}{c} E[\Delta X\Delta X^{⊤}|X(t)=X]=\sum_{k=1}^{K}\lambda_{k}(X)\;\nu_{k}\nu_{k}^{⊤}\;dt+o(dt). \end{array}$$

Therefore,


$$\begin{array}{cc} Cov(\Delta X|X(t)=X) & =E[\Delta X\Delta X^{⊤}|X]-E[\Delta X|X]E[\Delta X|X{]}^{⊤}\\ & =\sum_{k=1}^{K}\lambda_{k}(X)\;\nu_{k}\nu_{k}^{⊤}\;dt+o(dt), \end{array}$$ (55)

since the outer product of the conditional mean is *O*(*dt*^2^).

Hence, the exact infinitesimal covariance per unit time at the count scale is


$$\begin{array}{c} G(X):=\lim_{dt↓0}\frac{1}{dt}\;Cov(\Delta X|X(t)=X)=\sum_{k=1}^{K}\lambda_{k}(X)\;\nu_{k}\nu_{k}^{⊤}=S\;diag(\lambda (X))\;S^{⊤}. \end{array}$$ (56)

This identity is exact for the CTMC and is the source of the diffusion covariance used in the chemical Langevin approximation.

#### Density-dependent scaling and the diffusion matrix.

Introduce a system-size parameter Ω≫1 and define density variables


$$\begin{array}{c} x:=\frac{X}{\Omega }\in R_{+}^{d}. \end{array}$$

Assume density-dependent propensities of Kurtz type:


$$\begin{array}{c} \lambda_{k}(X)=\Omega \;f_{k}\;\left(\frac{X}{\Omega }\right)=\Omega \;f_{k}(x),\;k=1,\ldots ,K, \end{array}$$ (57)

for smooth rate functions $f_{k}:R_{+}^{d}\to R_{+}$. Let $f(x)=(f_{1}(x),\ldots ,f_{K}(x){)}^{⊤}$.

Under the standard chemical Langevin scaling, the density process is approximated by a diffusion with drift and covariance


$$\begin{array}{c} b(x)=S\;f(x),\;a(x)=\frac{1}{\Omega }\;S\;diag(f(x))\;S^{⊤}. \end{array}$$ (58)

The same formula follows directly by rescaling the CTMC short-time covariance (55): since Δx=ΔX/Ω and x=X/Ω,


$$\begin{array}{cc} Cov(\Delta x|x) & =\frac{1}{\Omega^{2}}\;Cov(\Delta X|X)\\ & =\frac{1}{\Omega^{2}}\left(\sum_{k=1}^{K}\lambda_{k}(X)\;\nu_{k}\nu_{k}^{⊤}\;dt\right)+o\;\left(\frac{dt}{\Omega }\right)\\ & =\frac{1}{\Omega }\left(\sum_{k=1}^{K}f_{k}(x)\;\nu_{k}\nu_{k}^{⊤}\right)dt+o\;\left(\frac{dt}{\Omega }\right)\\ & =a(x)\;dt+o\;\left(\frac{dt}{\Omega }\right). \end{array}$$ (59)

Thus, the diffusion matrix *a*(*x*) is precisely the density-scale counterpart of the exact count-scale infinitesimal covariance *G*(*X*).

### Proofs for well-posedness

This appendix provides detailed proofs of the main well-posedness statements in Well-posedness subsection in Methods section. We work on the open state space


$$\begin{array}{c} D=(0,\infty {)}^{2}\subset R^{2}, \end{array}$$

and consider the Itô SDE


$$\begin{array}{c} dx_{t}=b(x_{t})\;dt+\Sigma (x_{t})\;dW_{t},\;x_{0}\in D, \end{array}$$ (60)

where *W* is an *r*-dimensional Brownian motion and $a(x)=\Sigma (x)\Sigma (x{)}^{⊤}$.

For convenience, we restate the generator acting on $V\in C^{2}(D)$:


$$\begin{array}{c} LV(x)=\nabla V(x)\cdot b(x)+\frac{1}{2}tr\;(a(x)\nabla^{2}V(x)). \end{array}$$ (61)

We also recall the first boundary-hitting time


$$\begin{array}{c} \tau_{\partial D}:=\inf\{t\geq 0:\;x_{t}\notin D\}. \end{array}$$ (62)

#### 7.3.1. Preliminaries: exhaustion, localization, and a geometric lemma.

We begin with standard localization objects and a geometric fact used in the proof of Theorem 0.6.

We detail exhaustion of *D* by precompact sets. Fix an increasing exhaustion $(K_{n}{)}_{n\geq 1}$ of *D* by compact sets with nonempty interior such that


$$\begin{array}{c} K_{n}\subset \mathrm{int}(K_{n+1}),\;K_{n}⋐D,\;\overset{\infty }{\underset{n=1}{⋃}}K_{n}=D. \end{array}$$ (63)

A concrete choice is


$$\begin{array}{c} K_{n}:=[\frac{1}{n},n{]}^{2}\cap \overset{―}{B(0,n)}. \end{array}$$

For a continuous adapted process *x*, define the exit times


$$\begin{array}{c} \tau_{n}:=\inf\{t\geq 0:\;x_{t}\notin K_{n}\}. \end{array}$$ (64)

Then $\tau_{n}$ is increasing in *n*, and we set


$$\begin{array}{c} \tau_{*}:=\lim_{n\to \infty }\tau_{n}\in (0,\infty ]. \end{array}$$ (65)

**Lemma 0.8** (Leaving every compact subset of *D* while norm-bounded implies boundary approach). *Let* $D=(0,\infty {)}^{2}$*. Suppose* $(x_{n}{)}_{n\geq 1}\subset D$ *satisfies*


$$\begin{array}{c} \sup_{n\geq 1}\|x_{n}\|<\infty . \end{array}$$

*Furthermore, for every compact K⋐D, only finitely many x_n_ lie in K. Then*


$$\begin{array}{c} \mathrm{dist}(x_{n},\partial D)\to 0\;\text{along a subsequence}. \end{array}$$

*Equivalently, there exists a subsequence $\left(x_{n_{j}}\right)$ with $x_{n_{j}}\to x_{\infty }\in \partial D$ after passing to a further subsequence.*

*Proof.* Since (*x*_*n*_) is norm-bounded in $R^{2}$, Bolzano–Weierstrass theorem yields a convergent subsequence $x_{n_{j}}\to x_{\infty }\in \overset{―}{D}=[0,\infty {)}^{2}$. Suppose, for contradiction, that $x_{\infty }\in D$. Then there exists ε>0 such that $\overset{―}{B(x_{\infty },\varepsilon )}\subset D$. By convergence, for sufficiently large *j* we have $x_{n_{j}}\in \overset{―}{B(x_{\infty },\varepsilon )}$, so the infinite tail $\left\{x_{n_{j}}:j\geq J\right\}$ is contained in the compact set $\overset{―}{B(x_{\infty },\varepsilon )}⋐D$. This contradicts the standing assumption that for every compact K⋐D only finitely many terms of $\left(x_{n}\right)$ belong to *K*. Hence $x_{\infty }\in \partial D$, which implies $\mathrm{dist}(x_{n_{j}},\partial D)\to 0$. □

#### 7.3.2. Complete proof of Theorem 0.6.

*Proof.* The proof is divided into five steps.

**Step 1: Local strong solutions on each localization domain.** Fix *n* ≥ 1. By assumption **(A1)**, the coefficients b,Σ are Lipschitz and bounded on $K_{n}⋐D$. Extend b,Σ to globally Lipschitz, bounded coefficients $b^{\left(n\right)},\Sigma^{\left(n\right)}$ on $R^{2}$ such that


$$\begin{array}{c} b^{\left(n\right)}(x)=b(x),\;\Sigma^{\left(n\right)}(x)=\Sigma (x)\;\text{for }x\in K_{n}. \end{array}$$

(For example, use a cutoff and McShane-type extension componentwise.) Then the globally defined SDE


$$\begin{array}{c} dX_{t}^{\left(n\right)}=b^{\left(n\right)}(X_{t}^{\left(n\right)})\;dt+\Sigma^{\left(n\right)}(X_{t}^{\left(n\right)})\;dW_{t},\;X_{0}^{\left(n\right)}=x_{0}, \end{array}$$ (66)

admits a unique global strong solution by standard Itô SDE theory.

Define the exit time from *K*_*n*_ for this solution:


$$\begin{array}{c} \tau_{n}^{\left(n\right)}:=\inf\{t\geq 0:\;X_{t}^{\left(n\right)}\notin K_{n}\}. \end{array}$$

On $\left[0,\tau_{n}^{\left(n\right)}\right)$, $X_{t}^{\left(n\right)}\in K_{n}$, so *b*^(*n*)^ = *b* and $\Sigma^{\left(n\right)}=\Sigma$. Therefore *X*^(*n*)^ solves the original SDE (60) up to $\tau_{n}^{\left(n\right)}$.

**Step 2: Consistency and pathwise uniqueness patching.** Let *m* > *n*. Consider the extended solutions *X*^(*n*)^ and *X*^(*m*)^ driven by the same Brownian motion and the same initial condition. On *K*_*n*_, both sets of extended coefficients agree with the original coefficients (b,Σ), and **(A1)** yields local Lipschitz continuity on *K*_*n*_. By pathwise uniqueness for locally Lipschitz SDEs up to the exit time from *K*_*n*_, we have


$$\begin{array}{c} X_{t}^{\left(n\right)}=X_{t}^{\left(m\right)}\;\text{for all }t<\tau_{n}^{\left(n\right)}\wedge \tau_{n}^{\left(m\right)}\;\text{a.s.} \end{array}$$

Hence the stopped processes are consistent across *n*, and we may define a process *x*_*t*_ on $t<\tau_{*}$ by patching:


$$\begin{array}{c} x_{t}:=X_{t}^{\left(n\right)}\;\text{whenever }t<\tau_{n}, \end{array}$$

where $\tau_{n}$ is the (common) exit time from *K*_*n*_ under the patched process. This yields a strong solution of (60) on $\left[0,\tau_{*}\right)$, with


$$\begin{array}{c} \tau_{*}=\lim_{n\to \infty }\tau_{n}. \end{array}$$

By the same local uniqueness argument, this maximal solution is pathwise unique up to $\tau_{*}$.

**Step 3: A Lyapunov estimate up to bounded-radius localization.** Fix *R* > 0 and define


$$\begin{array}{c} \sigma_{R}:=\inf\{t\geq 0:\;\|x_{t}\|\geq R\}\wedge \tau_{\partial D}. \end{array}$$ (67)

For each *R*, the stopped process $x_{t\wedge \sigma_{R}}$ remains in the bounded set $D\cap \overset{―}{B(0,R)}$, so Itô’s formula applies to $V_{\infty }(x_{t\wedge \sigma_{R}})$. Using (61) and assumption **(A2)**,


$$\begin{array}{c} V_{\infty }(x_{t\wedge \sigma_{R}})=V_{\infty }(x_{0})+\int_{0}^{t\wedge \sigma_{R}}LV_{\infty }(x_{s})\;ds+M_{t\wedge \sigma_{R}}\\ \leq V_{\infty }(x_{0})+C\int_{0}^{t\wedge \sigma_{R}}(1+V_{\infty }(x_{s}))\;ds+M_{t\wedge \sigma_{R}}, \end{array}$$ (68)

where $M_{t\wedge \sigma_{R}}$ is a martingale with zero expectation. Taking expectations gives


$$\begin{array}{c} E[V_{\infty }(x_{t\wedge \sigma_{R}})]\leq V_{\infty }(x_{0})+Ct+C\int_{0}^{t}E[V_{\infty }(x_{s\wedge \sigma_{R}})]\;ds. \end{array}$$ (69)

By Grönwall’s inequality,


$$\begin{array}{c} E[V_{\infty }(x_{t\wedge \sigma_{R}})]\leq e^{Ct}(V_{\infty }(x_{0})+Ct),\;t\geq 0. \end{array}$$ (70)

The right-hand side is independent of *R*.

**Step 4: No explosion to infinity before boundary hitting.** We show that the process cannot lose well-posedness in the interior by escaping to infinity before hitting ∂D. Suppose, for contradiction, that there exists *T* > 0 such that


$$\begin{array}{c} P(\tau_{*}\leq T,\;\tau_{*}<\tau_{\partial D},\;\sup_{t<\tau_{*}}\|x_{t}\|=\infty )>0. \end{array}$$

On the event inside the probability, $\sigma_{R}\leq \tau_{*}\wedge T$ for all sufficiently large *R*, and $\|x_{\sigma_{R}}\|=R$. Since $\tau_{*}<\tau_{\partial D}$, the process remains in *D* up to $\tau_{*}$, so $\|x_{\sigma_{R}}\|\to \infty$ within *D*. By assumption **(A2)**,


$$\begin{array}{c} V_{\infty }(x_{\sigma_{R}})\to \infty \;\text{on that event.} \end{array}$$

Thus $V_{\infty }(x_{T\wedge \sigma_{R}})\to \infty$ on a set of positive probability. By Fatou’s lemma, $\sup_{R}E[V_{\infty }(x_{T\wedge \sigma_{R}})]=\infty$, contradicting the uniform estimate (70). Therefore,


$$\begin{array}{c} P(\tau_{*}<\infty ,\;\tau_{*}<\tau_{\partial D},\;\sup_{t<\tau_{*}}\|x_{t}\|=\infty )=0. \end{array}$$ (71)

**Step 5: If every compact set is eventually left while the norm remains bounded, the path approaches** ∂D.

It remains to rule out the possibility that


$$\begin{array}{c} \tau_{*}<\infty ,\;\tau_{*}<\tau_{\partial D},\;\sup_{t<\tau_{*}}\|x_{t}\|<\infty . \end{array}$$

On this event, since $\tau_{*}=\lim_{n\to \infty }\tau_{n}$ with $\tau_{n}=\inf\{t:\;x_{t}\notin K_{n}\}$ and $K_{n}⋐D$ increasing to *D*, the trajectory leaves each compact *K*_*n*_ before time $\tau_{*}$. Hence we may choose times $t_{n}↑\tau_{*}$ such that


$$\begin{array}{c} x_{t_{n}}\notin K_{n}. \end{array}$$

Because $\sup_{t<\tau_{*}}\|x_{t}\|<\infty$, the sequence $(x_{t_{n}})\subset D$ is norm-bounded. Moreover, for any compact K⋐D there exists *N* such that $K\subset K_{N}$; therefore $x_{t_{n}}\notin K$ for all *n* ≥ *N*. Thus $\left(x_{t_{n}}\right)$ is eventually outside every compact subset of *D*.

By Lemma 0.8, any norm-bounded sequence in *D* that eventually leaves every compact subset of *D* admits a subsequence whose distance to ∂D tends to zero. Consequently, some subsequence of $\left(x_{t_{n}}\right)$ approaches ∂D.

Since *x*_*t*_ is continuous on $\left[0,\tau_{*}\right)$, it follows that


$$\begin{array}{c} \underset{0\leq t<\tau_{*}}{\inf}\mathrm{dist}(x_{t},\partial D)=0, \end{array}$$

that is, the path approaches the boundary as $t↑\tau_{*}$.

This contradicts the event $\tau_{*}<\tau_{\partial D}$, which asserts that no boundary hit or exit occurs before time $\tau_{*}$. Hence any loss of compactness in *D* while the norm remains bounded must correspond to approach to the boundary rather than interior breakdown.

Combining this with (71), we conclude


$$\begin{array}{c} P(\tau_{*}<\infty ,\;\tau_{*}<\tau_{\partial D})=0. \end{array}$$

This completes the proof.

#### 7.3.3. Complete proof of Proposition 0.7.

*Proof.* Let (*K*_*n*_) and $\left(\tau_{n}\right)$ be the exhaustion and exit times defined in (63)–(64). By Theorem 0.6, a unique maximal strong solution exists up to $\tau_{*}$, and any finite lifetime obstruction can only occur through boundary hitting/approach.

**Step 1: Localized Itô estimate for the barrier Lyapunov function.** Fix *n* ≥ 1 and *t* ≥ 0. Since $x_{s\wedge \tau_{n}}\in K_{n}$ for all *s*, Itô’s formula for $V_{b}(x_{t\wedge \tau_{n}})$ yields


$$\begin{array}{c} V_{b}(x_{t\wedge \tau_{n}})=V_{b}(x_{0})+\int_{0}^{t\wedge \tau_{n}}LV_{b}(x_{s})\;ds+M_{t\wedge \tau_{n}}^{\left(b\right)}\\ \leq V_{b}(x_{0})+C_{b}\int_{0}^{t\wedge \tau_{n}}(1+V_{b}(x_{s}))\;ds+M_{t\wedge \tau_{n}}^{\left(b\right)}, \end{array}$$ (72)

where $M_{t\wedge \tau_{n}}^{\left(b\right)}$ is a true martingale.

Taking expectations,


$$\begin{array}{c} E[V_{b}(x_{t\wedge \tau_{n}})]\leq V_{b}(x_{0})+C_{b}t+C_{b}\int_{0}^{t}E[V_{b}(x_{s\wedge \tau_{n}})]\;ds. \end{array}$$ (73)

Applying Grönwall,


$$\begin{array}{c} E[V_{b}(x_{t\wedge \tau_{n}})]\leq e^{C_{b}t}(V_{b}(x_{0})+C_{b}t),\;\forall n\geq 1,\;\forall t\geq 0. \end{array}$$ (74)

Hence


$$\begin{array}{c} \sup_{n\geq 1}E[V_{b}(x_{t\wedge \tau_{n}})]<\infty \;\text{for each fixed }t\geq 0. \end{array}$$ (75)

**Step 2: Contradiction argument if boundary is hit with positive probability.** Assume for contradiction that $P(\tau_{\partial D}<\infty )>0$. Then there exists *T* > 0 such that


$$\begin{array}{c} P(\tau_{\partial D}\leq T)>0. \end{array}$$

On the event $\left\{\tau_{\partial D}\leq T\right\}$, continuity of sample paths implies $x_{t}\to x_{\tau_{\partial D}}\in \partial D$ as $t↑\tau_{\partial D}$ from below (or equivalently $x_{t}\to \partial D$ within *D*). By the barrier blow-up property **(B1)**,


$$\begin{array}{c} V_{b}(x_{t})\to \infty \;\text{as }t↑\tau_{\partial D}\text{ on }\{\tau_{\partial D}\leq T\}. \end{array}$$

Because $\tau_{n}↑\tau_{*}$ and Theorem 0.6 implies $\tau_{*}\geq \tau_{\partial D}$ a.s., the stopping sequence $T\wedge \tau_{n}$ approaches $T\wedge \tau_{\partial D}$ from below on $\left\{\tau_{\partial D}\leq T\right\}$. In particular,


$$\begin{array}{c} V_{b}(x_{T\wedge \tau_{n}})\to \infty \;\text{on }\{\tau_{\partial D}\leq T\}. \end{array}$$ (76)

Applying Fatou’s lemma to the nonnegative random variables $V_{b}(x_{T\wedge \tau_{n}})1_{\left\{\tau_{\partial D}\leq T\right\}}$, we obtain


$$\begin{array}{cc} \infty & =E\;\left[{lim inf}_{n\to \infty }V_{b}(x_{T\wedge \tau_{n}})1_{\left\{\tau_{\partial D}\leq T\right\}}\right]\\ & \leq {lim inf}_{n\to \infty }E\;\left[V_{b}(x_{T\wedge \tau_{n}})1_{\left\{\tau_{\partial D}\leq T\right\}}\right]\leq {lim inf}_{n\to \infty }E[V_{b}(x_{T\wedge \tau_{n}})], \end{array}$$ (77)

contradicting the uniform bound (75) at time *T*.

Therefore $P(\tau_{\partial D}\leq T)=0$ for every *T* > 0, hence


$$\begin{array}{c} P(\tau_{\partial D}<\infty )=0. \end{array}$$

**Step 3: Globality and positivity invariance.** Since boundary hitting is a.s. impossible and Theorem 0.6 excludes interior breakdown before boundary hitting, the maximal solution extends for all times:


$$\begin{array}{c} \tau_{*}=\infty \;\text{a.s.} \end{array}$$

Moreover,


$$\begin{array}{c} x_{t}\in D\;\text{for all }t\geq 0\;\text{a.s.} \end{array}$$

This proves positivity invariance.

#### 7.3.4. Remarks on verifiability of the assumptions in the present model.

**Remark** 0.9 (Local regularity of *b* and *a* on *D*). *For the R–M demographic diffusion constructed from Mechanistic CTMC and CME subsection to CME-consistent covariance structure subsection in Methods section, the drift b(x) and covariance a(x) are polynomial/rational combinations of N,P with denominator 1 + N. Since N > 0 on* $D=(0,\infty {)}^{2}$*, the denominator never vanishes on D, and therefore b and a are smooth on D (in particular, locally Lipschitz on every* K⋐D*).*

**Remark 0.10** (Local regularity of common factorizations Σ). *Two coefficient factorizations used in the paper fit assumption* ***(A1)*** *on precompact subsets* K⋐D*:*

(i) *Event-based factorization. If*
  $$\begin{array}{c} \Sigma_{K}(x)=\frac{1}{\sqrt{\Omega }}\;S\;\sqrt{diag(f(x))}, \end{array}$$
  *then on* K⋐D*, each intensity* *f*_*j*_(*x*) *is smooth and bounded away from singularities; in the present model the relevant* *f*_*j*_ *are nonnegative smooth functions on D. Hence* $\sqrt{f_{j}(x)}$ *is locally Lipschitz on K (in particular when* *f*_*j*_ *is bounded away from 0, and more generally for the explicit polynomial/rational intensities used here on compacts).*
(ii) *Matrix square-root factorization. If* Σ(x) *is chosen as a measurable square root of a(x) (e.g., Cholesky where a(x) is positive definite), then one typically works locally on compact subsets on which rank/signature does not change. On such sets,* Σ *can be chosen locally Lipschitz. For the well-posedness statements in Well-posedness subsection in Methods section, only local Lipschitz regularity on each* K⋐D *is required.*

**Remark 0.11** (Why boundary degeneracy does not conflict with the open-domain theorem). *Demographic diffusions are often degenerate at* ∂D *because reaction intensities vanish as* N↓0 *and/or* P↓0*. This does not contradict assumption* ***(A1)****, which is imposed only on precompact subsets* K⋐D*, i.e., strictly away from the boundary. The boundary behavior is handled separately through the maximal-solution formulation and, when needed, the barrier-Lyapunov condition of Proposition 0.7.*

#### 7.3.5. Abstract lemma: Two-stage Lyapunov architecture on open domains.

The arguments above can be summarized in a reusable form for boundary-degenerate ecological diffusions on open domains.

**Lemma 0.12** (Two-stage Lyapunov template on an open domain). *Let* $D\subset R^{d}$ *be open, and consider*


$$\begin{array}{c} dX_{t}=b(X_{t})\;dt+\Sigma (X_{t})\;dW_{t},\;X_{0}\in D, \end{array}$$

*with coefficients locally Lipschitz and locally bounded on every precompact* K⋐D*. Assume:*

(a) *there exists* $V_{\infty }\in C^{2}(D;[0,\infty ))$ *with* $V_{\infty }(x)\to \infty$ *as* ‖x‖→∞ *in D, and*
  $$\begin{array}{c} LV_{\infty }\leq C_{\infty }(1+V_{\infty }); \end{array}$$
(b) *optionally, there exists* $V_{b}\in C^{2}(D;[0,\infty ))$ *with* $V_{b}(x)\to \infty$ *as* x→∂D *within D, and (locally on an exhaustion)*
  $$\begin{array}{c} LV_{b}\leq C_{b}(1+V_{b}). \end{array}$$

*Then:*

(i) *under (a), there exists a unique maximal strong solution, and no loss of well-posedness occurs in the interior before boundary hitting;*
(ii) *under (a)+(b), the boundary is a.s. not hit in finite time, hence the solution is global and remains in D for all* *t* ≥ 0.

*Proof.* Part (i) follows the localization/patching and *V*_∞_-Lyapunov estimate used in the proof of Theorem 0.6; part (ii) follows by the barrier contradiction argument used in the proof of Proposition 0.7.

Lemma 0.12 is not needed for any statement in the main text, but it clarifies the general structure of the two-stage Lyapunov architecture and may be useful for extensions to other demographic diffusions and food-web models.
